# RNAi-Based Identification of Gene-Specific Nuclear Cofactor Networks Regulating Interleukin-1 Target Genes

**DOI:** 10.3389/fimmu.2018.00775

**Published:** 2018-04-27

**Authors:** Johanna Meier-Soelch, Liane Jurida, Axel Weber, Doris Newel, Johnny Kim, Thomas Braun, M. Lienhard Schmitz, Michael Kracht

**Affiliations:** ^1^Rudolf Buchheim Institute of Pharmacology, Justus Liebig University Giessen, Giessen, Germany; ^2^Max-Planck-Institute for Heart and Lung Research, Bad Nauheim, Germany; ^3^Institute of Biochemistry, Justus Liebig University Giessen, Giessen, Germany

**Keywords:** IL-1, corepressor, coactivator, NF-κB, transcription, chromatin, shRNA screen

## Abstract

The potent proinflammatory cytokine interleukin (IL)-1 triggers gene expression through the NF-κB signaling pathway. Here, we investigated the cofactor requirements of strongly regulated IL-1 target genes whose expression is impaired in p65 NF-κB-deficient murine embryonic fibroblasts. By two independent small-hairpin (sh)RNA screens, we examined 170 genes annotated to encode nuclear cofactors for their role in *Cxcl2* mRNA expression and identified 22 factors that modulated basal or IL-1-inducible *Cxcl2* levels. The functions of 16 of these factors were validated for *Cxcl2* and further analyzed for their role in regulation of 10 additional IL-1 target genes by RT-qPCR. These data reveal that each inducible gene has its own (quantitative) requirement of cofactors to maintain basal levels and to respond to IL-1. Twelve factors (*Epc1, H2afz, Kdm2b, Kdm6a, Mbd3, Mta2, Phf21a, Ruvbl1, Sin3b, Suv420h1, Taf1*, and *Ube3a*) have not been previously implicated in inflammatory cytokine functions. Bioinformatics analysis indicates that they are components of complex nuclear protein networks that regulate chromatin functions and gene transcription. Collectively, these data suggest that downstream from the essential NF-κB signal each cytokine-inducible target gene has further subtle requirements for individual sets of nuclear cofactors that shape its transcriptional activation profile.

## Introduction

Inflammation is an evolutionary conserved reaction to the myriad of insults that can affect tissue homeostasis. At the cellular level, the inflammatory reaction is characterized by rapid reprogramming of gene expression. The transiently expressed genes encode multiple factors that regulate immune cell infiltration and activation, blood vessel tone, metabolic reactions, pain and wound repair (1).

The founding members of the IL-1 family of cytokines, IL-1α and IL-1β, are master coordinators of inflammation as they can induce the full spectrum of clinical signs of inflammation by upregulating the entire repertoire of inflammatory genes in most (if not all) cell types of the body during sterile or infectious inflammation (2, 3).

IL-1 binding to its heterodimeric receptor at the plasma membrane triggers the cytoplasmic activation of the canonical NF-κB pathway and activation of JNK and p38 MAP kinases through a series of protein:protein interactions that require phosphorylations and ubiquitinylation events via linear and branched K63-ubiquitin chains (4).

Subsequently, promoter (and enhancer) binding of transcription factors (TFs) of the NF-κB and AP-1 families that are substrates of the NF-κB and MAPK signaling pathways trigger transcriptional induction of inflammatory genes through initiation of RNA polymerase II transcription cycles on each individual inflammatory target gene (5, 6).

In general, the quantitative and timely expression of genes requires further nuclear factors that are organized as large corepressor or coactivator multiprotein complexes to support, augment, or shut-down transcription (7, 8). These complexes possess enzymatic activity to modify N-terminal histone tails for regulating chromatin accessibility but also serve as scaffolding platforms to promote or repress stimulus- and tissue-specific activators of the transcription cycle (9).

Loss-of-function screens based on the RNA interference (RNAi) technology are powerful approaches to explore the function of mammalian genes at a large scale (10, 11). Mainly by using transiently transfected small interfering (si)RNAs, several groups have identified across different species signaling components that regulate transcriptional activity of NF-κB in response to a variety of immune stimuli including TNFα, a cytokine with inflammatory functions related to IL-1 (12–22). However, all these studies used artificial reporter systems to measure NF-κB activation as readouts for the RNAi screens, resulting in surprisingly little overlap of identified NF-κB modulators. The stable integration of NF-κB *cis*-elements upstream of luciferase or GFP reporter genes facilitates rapid, quantitative and high content assay screening but it gives little information on nuclear factors that regulate endogenous genes in their native chromatin environment (23). Accordingly, there is a lack of functional studies describing the full repertoire of coactivator/corepressor complexes in a given biological context, including the IL-1-driven inflammatory cell reaction.

Here, we report results from a small-hairpin (sh)RNA-based screen with selected regulators of chromatin organization and transcription that allowed the identification of 22 nuclear cofactors with differential effects on basal or IL-1-induced mRNA expression of 11 endogenous highly regulated NF-κB target genes. These data show that inflammatory genes require a gene-specific network of defined and interacting cofactors in addition to the canonical cytoplasmic signaling pathways that switch on their transcription.

## Results

### Considerations and Design of a shRNA Screen for Cofactors of IL-1 Target Gene Expression

**Figure 1:**
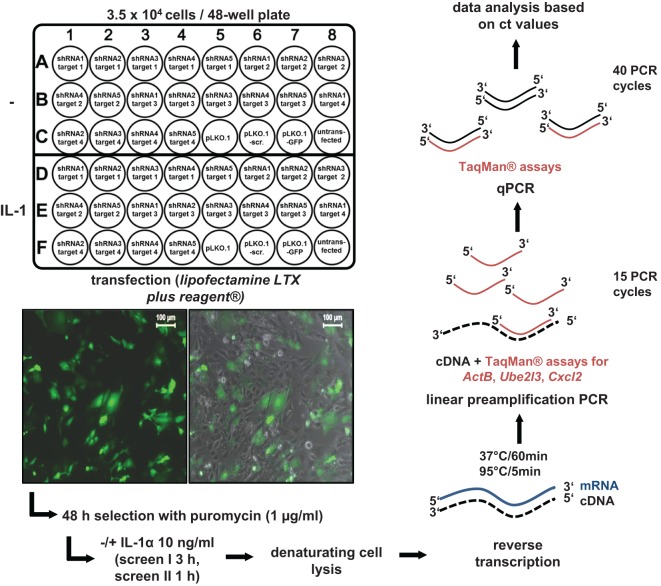
Design and execution of the small-hairpin (sh)RNA screens for murine nuclear cofactors of IL-1 signaling. For screen I, 4–5 shRNAs directed at up to four different nuclear targets per 48-well plate were transfected in duplicates as shown. In screen II, 4–5 shRNAs per nuclear target were pooled and transfected into a single well resulting in screening of 20 nuclear targets per plate (not shown). In both screens, empty vector (pLKO.1) with no insert or an insert encoding a scrambled shRNA sequence (pLKO.1-scr.) were used as controls on each of the plates. Cells transfected with pLKO.1 encoding a GFP cDNA were used to monitor transfection efficiency on each individual plate by fluorescence microscopy (left image) and by phase contrast plus fluorescence microscopy (right image) as shown by the insets. The scale bar is 100 µm. For each screen, 3.5 × 10^4^ cells were seeded per well. One day later, 270 ng of DNA were transfected using Lipofectamine LTX plus reagent^®^. Cells were selected for 48 h in 1 µg/ml puromycin. Then, half of the cells on each plate were left untreated. The other half was stimulated for 3 h (screen I) or 1 h (screen II) with IL-1α (10 ng/ml). Thereafter, cells were lysed, and RT reactions and preamplifications of cDNAs were performed using the PreAmp Cells-to-Ct_TM_ Kit and gene specific primers for the IL-1-inducible target gene *Cxcl2* and the two “housekeeping” genes *ActB* (screen I only) and *Ube2l3*. Finally, preamplified PCR products were subjected to quantitative (q)PCR using Taqman assays and mRNA expressions levels were quantified based on their cycle threshold (ct) value using an ABI7500 instrument. Ct values were used for all further calculations.

We developed a shRNA-based screen to identify nuclear coactivators or corepressors that modulate an endogenous IL-1-dependent gene response. Our set up combined (i) the knockdown of endogenous proteins classified as nuclear cofactors and (ii) the analysis of the resulting phenotype at the level of mRNA expression of an endogenous IL-1 target gene, thus avoiding the introduction of reporter genes or other means of further (genetic) manipulation. We initially searched microarray data sets of different cell types of human or murine origin for IL-1-mediated activation which led to the identification of a common core set of strongly regulated inflammatory genes thereby providing suitable targets as read out for our screening pipeline. In murine embryonic fibroblasts (Mefs), microarray experiments revealed *Cxcl2, Cxcl1, Cxcl5, Cxcl10, Ccl7, Ccl2, Ccl20, Il6, Nfkbia, Nfkbiz*, and *Icam1* as strongly regulated genes that were induced after 30 min of IL-1 stimulation (Figure S1A in Supplementary Material). We also screened a number of microarrays to identify genes that are not regulated by IL-1. We found no detectable changes for *ActB, Ube2l3, Ev1, Mcm7*, and *Car13* in response to IL-1, as confirmed by RT-qPCR experiments (Figure S1B in Supplementary Material). These data revealed the usability of these genes as internal (“housekeeping”) controls for loading of equal RNA amounts in PCR reactions (Figure S1B in Supplementary Material). RT-qPCR experiments for 11 of the IL-1 target genes confirmed that they were maximally induced between 30 and 180 min of IL-1 stimulation suggesting their onset of gene transcription might be coordinated by common nuclear mechanisms or cofactors (Figure S2 in Supplementary Material). We then selected a total of 170 well-annotated nuclear cofactors that had been described by the Kouzarides and Shen groups (24, 25). The full list of the known nuclear functions according to Panther/GO classifications of these proteins is shown in Table S1 in Supplementary Material. To suppress the protein levels of these coactivators, 791 plasmid vectors prepared from the TRC.1 shRNA library based on the vector pLKO.1-puro were transfected into primary Mefs (26). Notably, each nuclear cofactor encoding gene was targeted multiple times and individually (791/170 = 4.65 shRNAs/gene on average, Table S1 in Supplementary Material) enabling higher probability of identifying true positive hits when knockdowns gave rise to similar phenotypic changes. All screens were performed in a 48-well format, each well containing initially 3.5 × 10^4^ Mef cells. Transfection was performed with 270 ng plasmid DNA using lipofectamine LTX plus reagent under optimized conditions. Transfection rates were high as monitored using a GFP plasmid. In addition, cells were also selected with puromycin to eliminate the remaining non-transfected cells (Figure 1). On each plate, cells were also transfected with two different control plasmids, i.e., empty pLKO.1 or pLKO.1 containing a scrambled shRNA sequence. Then, cells were treated with IL-1 for 3 h (screen I) or 1 h (screen II) or were left untreated and reverse transcription was performed in denatured total cell extracts following an adapted commercial protocol. To amplify specific cDNAs, a linear preamplification PCR of 15 cycles was included using a pool of primer pairs contained in commercial Taqman assay kits. Aliquots of these cDNA mixtures were then further amplified, detected, and quantified by gene-specific quantitative PCR using the Taqman probes/primer pairs (Figure 1).

### Overview of Screening Results and Determination of shRNA Screening Hits

**Figure 2:**
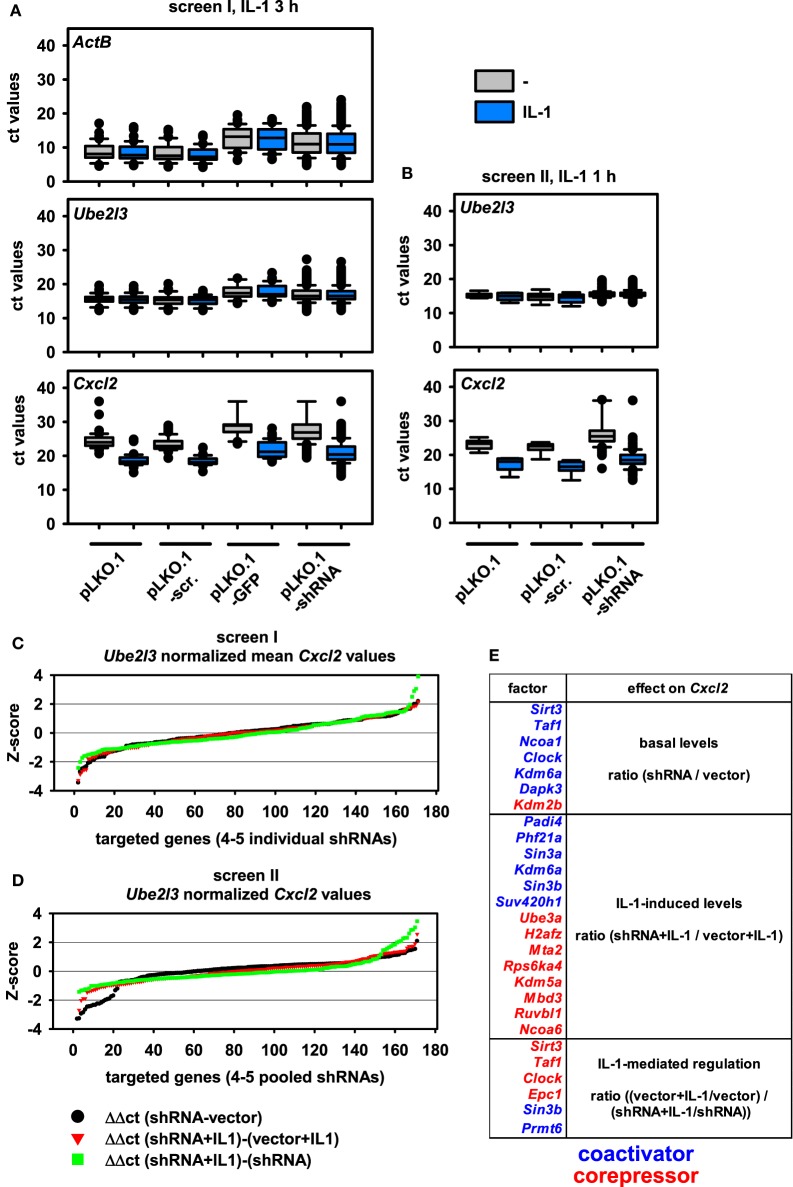
Distribution of mRNA measurements and changes of mRNA expression levels by shRNA-mediated knockdown of 170 nuclear cofactors using 4–5 transfected shRNAs per target. **(A)** For screen I, murine embryonic fibroblast (Mef) cells were transfected with 791 shRNA vectors representing 170 nuclear cofactors on a total of 43 plates according to the scheme shown in Figure 1. On each plate, empty pLKO.1, pLKO.1 encoding a scrambled shRNA, and pLKO.1 encoding GFP were transfected as controls. After 48 h of selection in puromycin, half of the cells per plate were left untreated (-, gray boxes) or were stimulated with IL-1 (blue boxes) for 3 h and mRNA measurements for *ActB, Ube2l3*, and *Cxcl2* were performed as described in the legend of Figure 1. Box plots show the distribution of all cycle threshold (ct) values obtained. **(B)** A repeat experiment (screen II) was performed under identical conditions with the two following modifications: (i) the shRNA encoding vectors for each of the 170 nuclear targets were combined and the pool was transfected into a single well on a total of 9 plates and (ii) cells were stimulated with IL-1 for 1 h. Box plots show the distribution of all ct values obtained. **(C)** For all target genes analyzed in screen I, individual ct values obtained for *Cxcl2* were normalized to the ct values of *Ube2l3*. The resulting Δct values of the 4–5 shRNAs per target were averaged and used to calculate differences in mRNA expression levels between untreated or IL-1-stimulated cells and relative to the vector (pLKO.1)-transfected control cells as shown by the equations. The graph shows the ranked Z-scores of the resulting ΔΔct values. **(D)** An identical analysis was performed and visualized by ranked Z-scores of ΔΔct values obtained from screen II. **(E)** Combined summary from both screens showing the nuclear cofactors that affect *Cxcl2* mRNA expression in untreated cells (basal level) or in IL-1-treated cells (IL-1 levels) or the extent of IL-1-mediated regulation (IL-1 regulation) as compared to the vector control cells. The selection is based on shRNA transfections resulting in a Z-score > or < 1 SD in both screens. Colors indicate the direction of the shRNA effect (blue suppression, red induction of mRNA levels) thereby defining the role of the downregulated nuclear factor as coactivator or corepressor.

Using these setups, we performed two independent shRNA screens using the most strongly regulated IL-1 target gene *Cxcl2* as a readout (Figure S1A in Supplementary Material). Apart from varying, the time of induction by IL-1 within the window of strong mRNA induction of *Cxcl2* (60 and 180 min) as defined by the experiments shown in Figure S2 in Supplementary Material, we also varied the number of different shRNAs transfected per well. In the first screen, all the different shRNAs available per gene were transfected individually, and single ct values were obtained. In the second screen, all shRNAs per gene were pooled and transfected together into the same well resulting in a single ct value for further calculations. Figure 2A shows the distribution of all RT-qPCR measurements obtained for screen I. The analysis of the medians and distribution of ct values of both control genes (*ActB, Ube2l3*) showed that there were little differences between control transfections (pLKO.1, pLKO.1-scr.) or IL-1 treatment as expected (Figures 2A,B). However, expression of GFP from the pLKO.1 vector backbone had a global suppressive effect on all *ActB* and *Ube2l3* ct values showing that this condition was useful for monitoring transfection efficiency on the individual 48 well plates, but not for normalization or further analyses of changes in gene expression (Figure 2A). Compared to vector controls, shRNA transfections lowered the median *ActB* mRNA level in screen I (Figure 2A). In contrast, there were little effects of the shRNAs on the median *Ube2l3* mRNA levels (Figures 2A,B). Therefore, we omitted *ActB* measurements as an internal control for the second screen and for all subsequent calculations of normalized mRNA levels. The IL-1 target gene *Cxcl2* was expressed at lower abundance (i.e., higher ct values) in unstimulated cells but was strongly regulated after exposure of cells to IL-1 as expected (Figure 2A). This was also the case for screen II (Figure 2B). As the goal of the screen was to identify changes of basal or inducible *Cxcl2* expression levels by knockdown of nuclear cofactors, it was important to consider a suitable control situation for relative quantification of qPCR data. As shown in Figures 2A,B, cells transfected with empty pLKO.1 or the scrambled shRNA show a comparable regulation of *Cxcl2* by IL-1, whereas expression of GFP reduced basal and inducible levels of *Cxcl2* (Figure 2A). This increase in ct values suggested that GFP also had a suppressive effect on IL-1-mediated gene expression. Therefore, we decided to choose pLKO.1 as (vector) control for calculating relative differences in mRNA expression levels. In both screens, transfection of shRNAs resulted in a global increase in median ct values for *Cxcl2* suggesting that multiple cofactors may be involved in regulating basal and inducible *Cxcl2* levels (Figures 2A,B).

We then considered various types of changes of *Cxcl2* mRNA levels that may occur in shRNA-treated cells. A schematic representation of equations reflecting these changes in basal conditions or in IL-1-treated cells as well as alterations in the extent of IL-1-mediated regulation in control cells versus shRNA-treated cells is shown in Figure S3 in Supplementary Material. The ΔΔct method was used to calculate these differences from ct values of *Cxcl2* after normalization to *Ube2l3* expression levels. The normalized values from screen I were merged to one expression (ct) value per shRNA target. Ratio values derived from the comparisons mentioned above were then ranked by Z-score (Figures 2C,D). Data from shRNAs with a Z-score value greater than 1 were then used to identify potential hits and to calculate the overlap of hits between screen I and screen II. To prove the validity of this selection procedure, individual fold changes for each shRNA target were visualized as heatmaps and are shown alongside all individual vector control values of the corresponding plates utilized in screens I and II (Figure S4 in Supplementary Material). With this strategy, 22 factors were identified whose suppression changed mRNA levels of *Cxcl2* significantly (i.e., <1 SD) in both screens (Figure 2E). Inspection of the data reveals that with a few exceptions (e.g., shKdm5a shRNA#4) the up to five shRNAs per gene changed ratios of *Cxcl2* mRNA expression into the same direction (Figure S4 in Supplementary Material). These observations underscore the reliability of the TRC.1 selection procedure for shRNAs and show that our strategy enables identification of true positive hits with high probability. Although the extent of *Cxcl2* regulation in the vector controls varied considerably between individual plates, the average regulation between both screens was comparable and showed strong induction by IL-1 (Figure S4 in Supplementary Material). We attribute this phenomenon to the relatively low basal mRNA expression level of *Cxcl2* and to the lack of controlling input RNA or cDNA concentrations across samples during the miniaturized RT-PCR amplification procedure (see Figure 1). These confounding factors may cause changes in calculated ratio values. However, the inspection of all data values shown in Figure S4 in Supplementary Material clearly revealed shRNAs that suppressed (blue colors) or induced (red colors) *Cxcl2* mRNA levels, thereby defining putative coactivators or corepressors of *Cxcl2* expression in untreated or IL-1-stimulated conditions as summarized in Figure 2E.

### Validation of Screening Hits and Identification of Positive or Negative Regulators of *Cxcl2*

**Figure 3:**
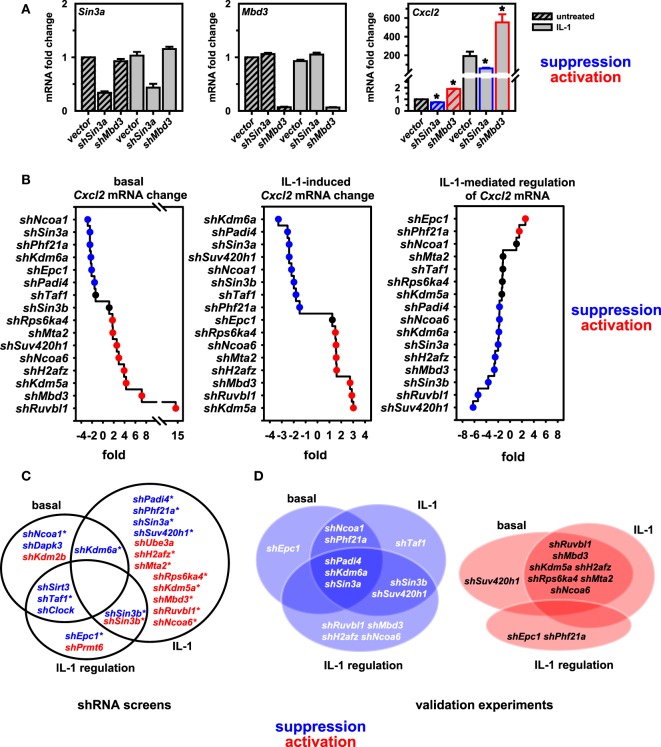
Validation of screen results further defines coactivators and corepressors of *Cxcl2*. **(A)** Murine embryonic fibroblast (Mef) cells were transfected with empty pLKO.1 or with pooled shRNAs directed against *Sin3a* or *Mbd3*. After 48 h of selection in puromycin, cells were treated with IL-1 for 1 h or were left untreated. Then, total RNA was isolated and mRNA expression of *Cxcl2, Sin3a, Mbd3* and *Ube2l3* was determined by conventional RT-qPCR. Expression values of *Cxcl2, Sin3a*, or *Mbd3* were normalized for expression of *Ube2l3*. The graphs show mean relative expression values ± SEM relative to the vector control from two independent series of experiments performed in duplicates. Asterisks indicate significant changes (*p* < 0.05) derived from Mann-Whitney Rank Sum *t*-tests. **(B)** The same RT-qPCR approach was used to determine the effects of 16 further knockdowns of nuclear cofactors on basal and IL-1-inducible *Cxcl2* levels as described in detail in the legend of Figure S5 in Supplementary Material. The line graphs show the ranked mean fold changes of normalized *Cxcl2* mRNA expression separately analyzed for basal and IL-1-induced conditions. Additionally, the effects on IL-1-mediated regulation (as defined in Figure S3 in Supplementary Material) are depicted. **(C)** Summarizing venn diagrams indicating the overlapping effects on *Cxcl2* mRNA expression of shRNAs directed against 22 nuclear cofactors as identified by shRNA screens I and II. Asterisks indicate the 16 factors that were chosen for validation. **(D)** Overlap of functions of the shRNAs as found by conventional RT-qPCR. **(A–D)** Red or blue colors indicate at least 1.5-fold differences between shRNA versus empty vector transfections.

To validate these findings, we performed conventional RT-qPCR experiments that allowed rigorous control of cell number, RNA purification and normalization for input total RNA in cDNA and qPCR reactions. Figure 3A demonstrates for two factors (*Sin3a* and *Mbd3*) the knockdown efficiency of pooled shRNAs and the suppressive or activating effect on basal and IL-1-inducible *Cxcl2* expression, confirming the results from the screen as shown in Figure S4 in Supplementary Material. We then extended this analysis initially focusing on nine putative coactivators and later on seven putative corepressors of *Cxcl2*, respectively. As these shRNA transfections were performed in two independent series of experiments with some time lag between them, we chose to keep them separated also for subsequent data analysis to report the data as faithful as possible. All *Cxcl2* mRNA expression data were normalized to *Ube2l3* and to the maximal IL-1-inducible levels in vector control cells. As shown in Figure S5 in Supplementary Material, these experiments confirmed a role of all factors in basal or inducible *Cxcl2* mRNA expression. Notable was a difference in the basal expression level of *Cxcl2* in the vector controls between experiment series (1) and (2) (Figure S5 in Supplementary Material). While this effect corroborates the biological variation of the low abundant *Cxcl2* transcript in cultured Mef cell lines, it does not affect the conclusion that knockdown of six factors (*Ncoa1, Sin3a, Phf21a, Kdm6a, Padi4, Epc1*) consistently suppressed basal *Cxcl2* levels, while knockdown of eight factors (*Suv420h1, Ruvbl1, Mbd3, Kdm5a, H2afz, Rps6ka4, Mta2, Ncoa6*) derepressed basal *Cxcl2* levels (Figure S5 in Supplementary Material). Knockdowns of all of these factors except for *Epc1* also affected the IL-1-induced mRNA level of *Cxcl2* by more than 1.5-fold (Figure S5 in Supplementary Material). The ranked effects of all knockdowns on *Cxcl2* levels are summarized in Figure 3B. 11 of the 16 factors are also needed as supporting factors for IL-1-mediated regulation of *Cxcl2* levels (Figure 3B, right panel). We define this signal-triggered difference as the ratio between basal and IL-1-induced levels (see Figure S3 in Supplementary Material). This ratio therefore reflects the IL-1 receptor-mediated signaling effect that activates *Cxcl2* transcription. Only two knockdowns (*shPhf21a, shEpc1*) further increase signaling because they regulate basal and inducible *Cxcl2* expression in opposite directions. Thereby these two factors can amplify the IL-1 response (Figure 3B, right panel). The overlapping hits between the combined screen results and the validation experiments are indicated by the Venn diagrams and are summarized in Figures 3C,D. By validation experiments, significant more factors were found to affect also the basal expression levels thus reducing the number of factors influencing IL-1 regulation compared to the screening results (Figures 3C,D). As outlined above, this can be attributed to the more reliable determination of changes in basal expression of *Cxcl2* by conventional RT-qPCR. Collectively, these data corroborate the validity of our screening pipeline and underscore the importance of the identified genes in regulating an IL-1 mediated inflammation response.

### Gene-Specific Requirements of the 16 Nuclear Cofactors for IL-1 Target Genes

**Figure 4:**
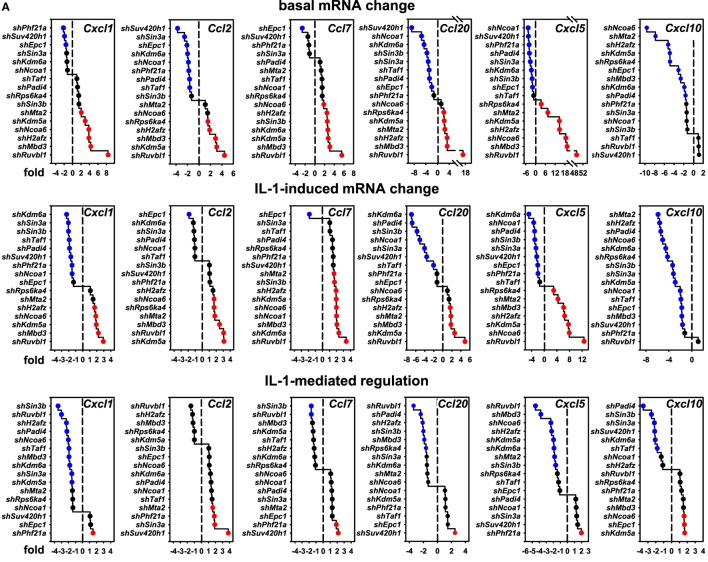

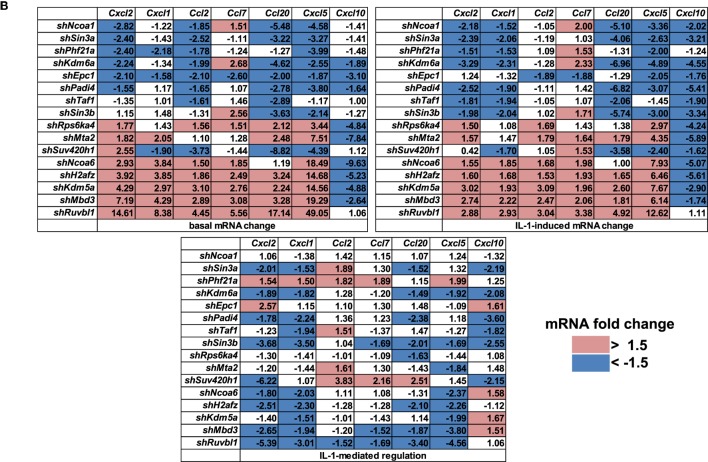
Gene-specific requirements of the 16 nuclear cofactors for 6 additional chemokine genes. **(A)** The same RNA preparations as described in Figure 3B were analyzed for the expression of six further chemokine genes. Shown are ranked fold changes for each mRNA. **(B)** Heatmaps visualizing the effects of individual knockdowns of cofactors across all genes. Data are ranked by the fold change of basal *Cxcl2* mRNA expression. Bar graphs representing all relative expression values are shown in Figure S6 in Supplementary Material. Red or blue colors indicate at least 1.5-fold differences between shRNA versus empty vector transfections.

**Figure 5:**
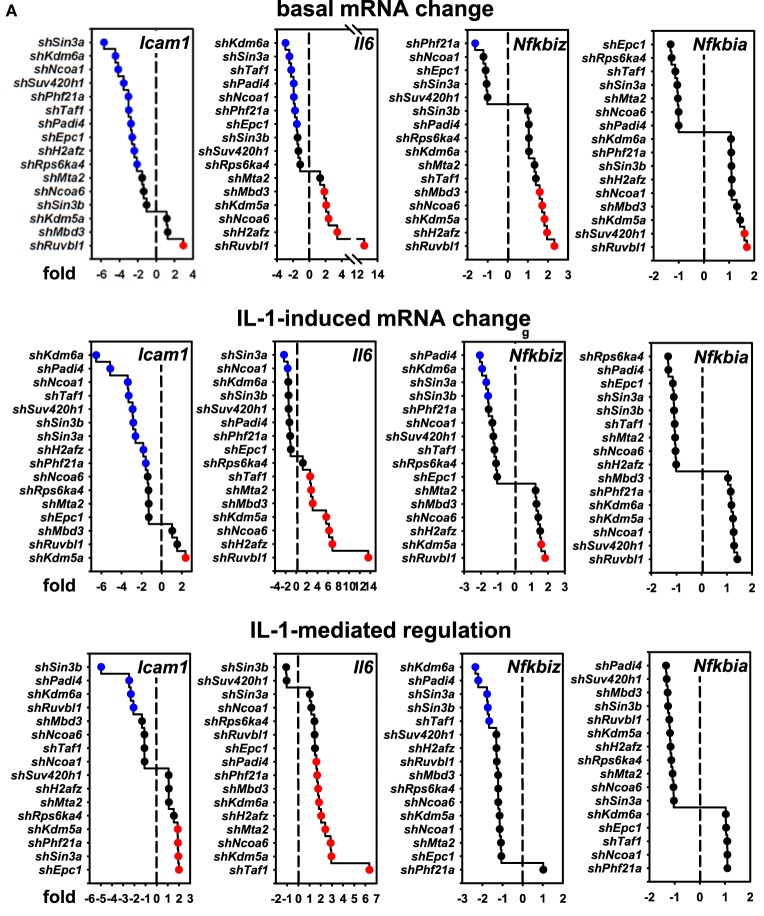

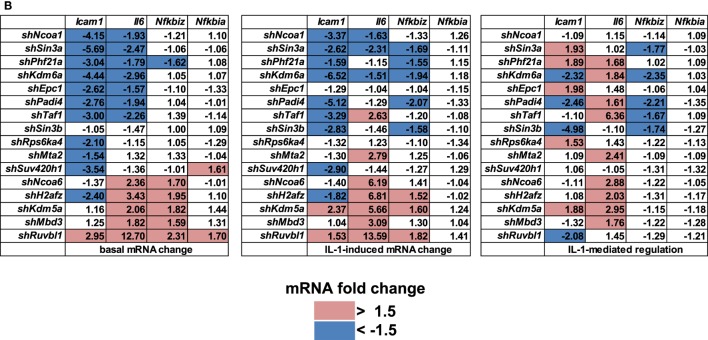
Gene-specific requirements of the 16 nuclear cofactors for 4 additional IL-1 target genes. **(A)** The same RNA preparations as described in Figure 3B were analyzed for the expression of four additional IL-1 targets representing adhesion molecules (*Icam1*), cytokines (*Il6*), and signaling regulators (*Nfkbiz, Nfkbia*). Shown are ranked fold changes for each mRNA. **(B)** Heatmaps visualizing the effects of individual knockdowns of cofactors across all genes. Data are ranked by the fold change of basal *Cxcl2* mRNA expression as in Figure 4B. Bar graphs representing all relative expression values are shown in Figure S7 in Supplementary Material. Red or blue colors indicate at least 1.5-fold differences between small-hairpin (sh)RNA versus empty vector transfections.

These results raised the question if these cofactors affected the IL-1 response in a gene-specific manner. We therefore tested the expression of ten additional IL-1 target genes using the same RNA preparations as described above (Figures S6 and S7 in Supplementary Material). Figure 4A summarizes the ranked changes in mRNA expression for six further chemokine genes located on different chromosomes (chr.1, *Ccl20;* chr. 5, *Cxcl1, Cxcl5, Cxcl10*; chr.11, *Ccl2, Ccl7*). These data reveal that each inducible gene has its own quantitative requirement of cofactors to maintain basal levels and to respond to IL-1. Figure 4B displays heatmaps to visualize how each cofactor affects mRNA levels across the seven chemokine genes tested including *Cxcl2* of chr. 5. Six factors (*Ncoa1, Sin3a, Phf21a, Kdm6a, Epc1, Padi4*) are required for basal and IL-1-induced expression for four out of seven chemokine genes; however, there are highly gene-specific variations. For example, *Ncoa1* and *Kdm6a* knockdowns suppress expression of *Cxcl2, Cxcl1, Ccl20, Cxcl5*, and *Cxcl10*, but enhance expression of *Ccl7*. Likewise, five factors (*Ncoa6, H2afz, Kdm5a, Mbd3, Ruvbl1*) suppress expression levels of six chemokine genes, but have opposite effects on *Cxcl10*. We also tested the effects of these knockdowns on four additional IL-1 target genes that have different biological functions (Figure 5A). Most remarkably, expression and regulation of the *Nfkbia* gene encoding the cytosolic IκBα inhibitor of NF-κB was almost unaffected by the knockdown of the 16 cofactors, whereas *Il6* and *Icam1* had similar positive cofactor requirements (compared to *Cxcl2*) but differed in the regulation by repressory genes (Figure 5B). A second inhibitor of NF-κB, *Nfkbiz*, varied significantly in the requirements of the 16 factors compared to all chemokine genes and to *Icam1* or *Il6* (Figure 5B). Bar graphs displaying the basal and inducible mRNA levels of all genes shown in Figures 4 and 5 are provided in Figures S6 and S7 in Supplementary Material. Re-analysis of data previously published by us reveals that basal and inducible expression of 10 of these genes is lost in p65 NF-κB-deficient Mefs but can be restored by reconstitution of this NF-κB subunit proving that this pathway provides the essential signals for their expression [Figure S8 in Supplementary Material; (27)]. *Ccl20* which was the only mRNA found not to be expressed in reconstituted Mefs was shown by others to be dependent on p65 (28). Evidence for direct regulation of the eleven genes by the NF-κB pathway is further provided by the enrichment of NF-κB elements in their promoters (Table S2 in Supplementary Material). An exemplary kinetic analysis for the knockdown of Sin3a confirmed the data obtained at the 1 h time point and also revealed that Sin3a functions as coactivator for *Cxcl2, Cxcl5, Cxcl10*, and *Ccl20*, but acts as a repressor of *Ccl7* and *Ccl2* (Figure 7). We also found that Sin3a was recruited to the promoter region of *Ccl2*, a gene which we previously showed to bind NF-κB p65 in an IL-1-dependent manner [Figure S9 in Supplementary Material; (29)].

**Figure 6:**
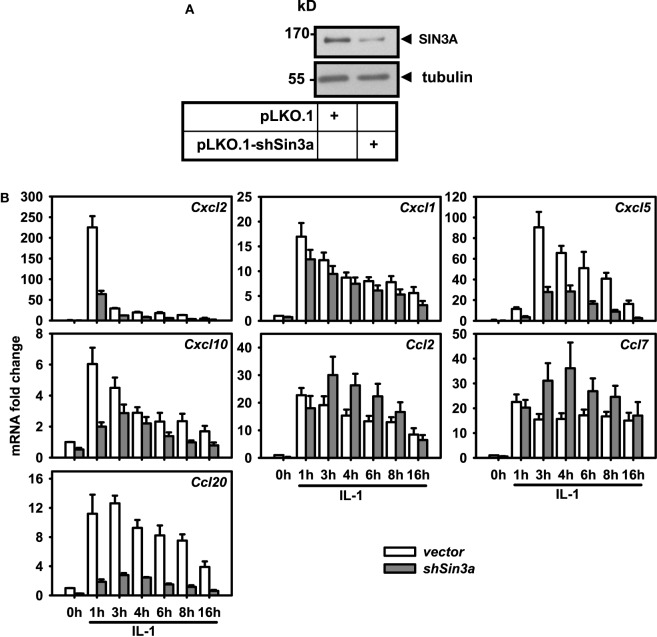
Differential effects of *Sin3a* knockdown on the expression of seven chemokine genes. Mef cells were transfected with pooled shRNAs directed against SIN3A (*shSin3a*) or with empty pLKO.1 (*vector*). After 48 h of selection in puromycin, cells were treated with IL-1 for the indicated times or were left untreated. **(A)** Expression of SIN3A was analyzed in total cell extracts by immunoblotting. Anti tubulin antibodies were used to confirm equal loading. **(B)** Total RNA was isolated and mRNA expression of the indicated chemokine genes and of *Ube2l3* was determined by conventional RT-qPCR. Expression values were normalized for expression of *Ube2l3*. The graph show relative expression values ± SEM. from at least two independent experiments.

**Figure 7:**
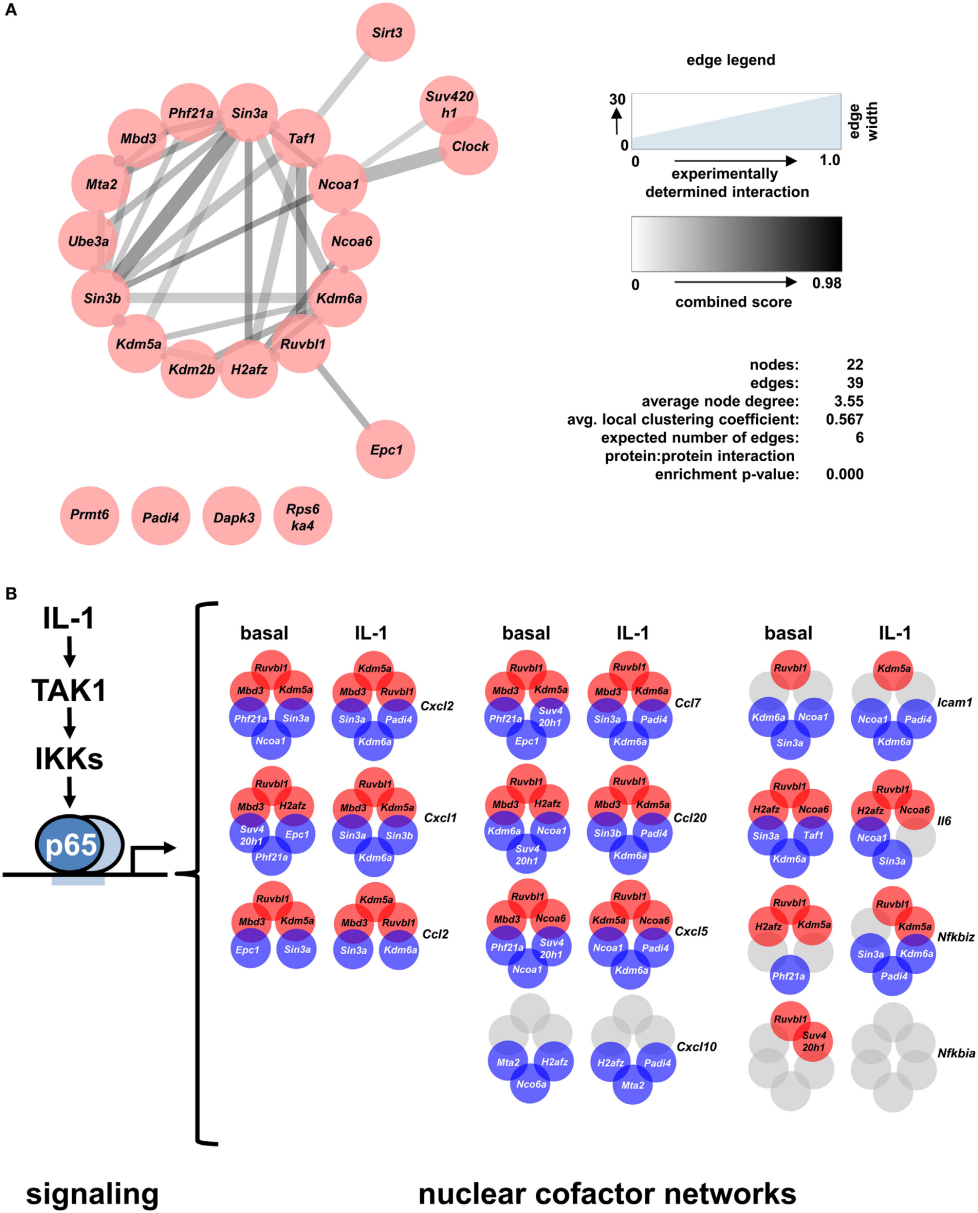
A differential set of closely interacting cofactors is required for basal or IL-1-inducible expression of *Cxcl2*. **(A)** The entire list of 22 nuclear cofactors as revealed by the combined analysis of both screens was analyzed for known protein:protein interactions of *mus musculus* using the STRING database (30) with the settings text mining, experiments, databases and coexpression, and a medium confidence score of 0.4. The resulting network was imported into Cytoscape and edges were visualized based on the experimental evidence for protein:protein interactions (edge width) and the STRING combined score (edge color). STRING network analysis also reveals that, compared to the whole genome, the nodes in the network show significantly more interactions than expected. **(B)** Summarizing scheme of the findings of this study which suggest that downstream of the canonical NF-κB signaling pathway each IL-1-response gene has its own nuclear cofactor requirement. Circles in red (corepressors) or blue (coactivators) colors show the three factors whose knockdowns most strongly activated or suppressed the basal or IL-1-inducible expression of eleven genes. Gray colors mark conditions in which knockdowns failed to deregulate mRNAs by more than 1.5 fold.

STRING database analysis revealed that 17 of the 22 identified factors are engaged in multiple protein:protein interactions based on experimental evidence, co-occurrence, and/or coexpression (Figure 7A) (30). They are predicted to form a multi protein network that shows many more interactions than expected compared to the whole genome as revealed by the network statistics (Figure 7A). Four factors have no yet known interactions within this network (Prmt6, Padi4, Dapk3, Rps6ka4). As the evidence contained in the STRING database reflects multiple biological conditions and species, we further asked if any of these factors have been previously implicated in IL-1, TNF, or NF-κB pathways. Table 1 summarizes the results from a detailed literature search indicating that 12 genes (*Epc1, H2afz, Kdm2b, Kdm6a, Mbd3, Mta2, Phf21a, Ruvbl1, Sin3b, Suv420h1, Taf1*, and *Ube3a*) have not been previously implicated in these responses. The others have some role in regulating NF-κB subunit function and some of them have also been related to TNF or IL-1 biology. However, a mechanistic role in regulation of IL-1 target genes has not been described so far for any of the 22 cofactors that we identified in this study. Thus, the experiments presented in Figures 4–7; Figures S5–S9 in Supplementary Material suggest that downstream of the NF-κB pathway each gene has further subtle requirements for nuclear cofactors that shape its transcriptional activation profile. Unlike NF-κB, however, none of these factors appears to be essential, but they are needed to fine tune mRNA expression levels. This hypothesis is summarized schematically in Figure 7B and provides a framework for future studies.

**Table 1:** Known functions and putative involvement of the 22 cofactors in IL-1, TNF, or NF-κB regulation.

	Nuclear Cofactor (gene symbol)	Alias	Prinicipal function	Implication in IL-1 signaling	Implication in TNF signaling	Implication in NF-κB transactivation	Species, cell types	Reference
1	*Clock*	BHLHE8, KAT13D	Heterodimeric TF factor (together with BMAL1), regulates circadian expression of genes	IL-1 suppresses Clock-dependent genes	TNF suppresses Clock-dependent genes; Clock enhances TNF-mediated transcription	Binds to p65, enhances NF-κB activity together with BMAL1	Mouse, human; fibroblasts, epithelial cells	(31–33)

2	*Dapk3*	ZIPK, DLK	Serine/threonine kinase involved in apoptosis, autophagy, transcription, IFNγ expression, granulocyte migration		Knockdown inhibits TNF-induced expression of VCAM-1		Rat; vascular smooth muscle cells (VSMC)	(34–36)

3	*Epc1*	Epl1	Enhancer of polycomb homolog 1, component of the NuA4 histone acetyltransferase (HAT) complex					(37, 38)

4	*H2afz*	H2AZ	Histone variant at promoters/TSS of active and poised genes					(39)

5	*Kdm2b*	JHDM1B, FBXL10, JEMMA	H3K4 and H3K36 demethylase, component of polycomb repressive complexes					(40, 41)

6	*Kdm5a*	JARID1A, RBBP2	H3K4 demethylase, enhances gene activation by CLOCK:BMAL1, required for IFNγ production of NK cells			Binds to p50 NF-κB	Mouse; splenocytes, NK cells	(42, 43)

7	*Kdm6a*	UTX, KABUK2	H3K27 demethylase, establishes active enhancers, involved in (auto)immune syndromes and T-cell activation					(44–46)

8	*Mbd3*		Binds (un)methylated CpG-rich active promoters and enhancers, component of the NuRD complex, essential for mouse development					(47, 48)

9	*Mta2*	MTA1L1,	component of NURD and remodeling complexes, binds to GATA3 in TH_2_ cells, suppresses IL-2, IL-4, IFNγ expression and autoimmunity					(49–51)

10	*Ncoa1*	BLHLE74, SRC1	Transcription coactivator of nuclear receptors		TNF-induced recruitment to IκBα promoter	Ectopically expressed protein enhances p65 transactivation and *Il6* promoter reporter activity	Rat, hamster; VSMC	(52, 53)

11	*Ncoa6*	RAP250, AIB3, ASC-2, TRBP	Transcription coactivator of nuclear receptors			Binds *in vitro* and by Y2H top 50/p65, activates NF-κB reporter	Mouse, human, yeast; fibroblasts, epithelial cells	(54)

12	*Padi4*	PADI-H	Protein-arginine deiminase, catalyzes the citrullination/deimination of histones/other proteins		Protein induced by TNF	No direct evidence	Mouse; VSMC	(55)

13	*Phf21a*	BHC80a	Interacts with and inhibits LSD1 demethylase activity, binds unmethylated H3K4 and COREST					(56–58)

14	*Prmt6*	HRMT1L6	Protein arginine methyltransferase, mediates H3R2, H3R42, H4R3, H2AR3 methylation		Enhances TNF-mediated expression of *IL6*, binds to the *IL6* promoter	Ectopic expression induces *IL6*, binds to p65 and activates NF-κB reporter	Mouse; tissues and Mefs	(59)

15	*Rps6ka4*	RSKB, MSK2, p90 RSK	Nuclear protein kinase downstream of ERK and p38 MAPK, phosphorylates H3S10, CREB1, ATF1, suppressor of inflammation	Increased levels of IL-1 in MSK1/2 ko mice in inflammed skin	Suppression of LPS-induced TNF secretion (together with MSK1)	Promotes phosphorylation of p65 at S276 and p65 transactivation, MSK1/2 ko increases skin inflammation	Mouse, human; macrophages, skin, epithelial cells, breast adenocarcinoma	(60–63)

16	*Ruvbl1*	TIP49A, TAP54-Alpha, INO80H	Chromatin remodeler and ATPase involved in nucleosome sliding					(64, 65)

17	*Sin3a*		Nuclear scaffold, transcriptional repressor, interacts with HDAC1/2 and multiple other proteins	Upregulation of *IL1A/B, CXCL1/3, IL6, IL8* by Sin3a knockdown in cells with activated PI3K		Binds to NF-κB subunits during the cell cycle^a^	Human; U2OS sarcoma cells, genetically modified Myr-PIK3 transformed ovarian cancer cells	(66–68)

18	*Sin3b*		Nuclear scaffold, transcriptional repressor, interacts with MYC and HDAC1/2, binds H3K4me3/H3K36me3-enriched nucleosomes					(69, 70)

19	*Sirt3*	SIR2L3	NAD-dependent class III (histone) deacetylase			Indirect through resveratol-mediated suppression of p65 translocation	Rat; cardiomyocytes	(71)

20	*Suv420h1*	KMT5B	methylates H4K20					(72, 73)

21	*Taf1*	TAFII250, NSCL2, CCG1	Component of TFIID, primary mediator of downstream promoter binding of the preinitiation complex					(74)

22	*Ube3a*	EPVE6AP, HPVE6A, E6AP	Ubiquitin E3 ligase, interacts with polycomb protein Ring1B, linked to the Angelman syndrome					(75, 76)

*^a^Reported in reference 67, a publication that has been retracted*.

## Discussion

Inflammatory cytokines execute their biological functions through the transcriptional regulation of specific sets of genes (5). Gene transcription is a highly coordinated process involving multiple molecular machines and their interactions with chromatin to regulate removal of the chromatin barrier, RNA polymerase II recruitment, pre-mRNA processing, editing, and export (77). Unlike the cytoplasmic signaling pathways which have been elucidated at a considerable level of detail, the (combinations of) nuclear factors that are required for IL-1-mediated gene regulation have largely been elusive (4). Here, we report the results from a limited shRNA screen revealing a novel network of nuclear cofactors that shape the cytokine-driven gene response of 11 inflammatory target genes. shRNA screens are powerful tools to unravel gene functions on a broad scale. Usually, therefore, they are performed using streamlined assays in an automatized way (10, 11). Here, we describe in detail a smaller categorized shRNA screen that can be performed by a single person using standard laboratory equipment. As a phenotypic readout, we chose to measure mRNA expression in response to IL-1. This more complicated multistep biological assay led us to limit the total number of investigated components to 170. By repeating the screen and by scoring the performance of individual shRNAs designed against the same target, we provide a thorough confirmation of the knockdown efficiency of individual shRNA constructs provided by the TRC consortium (26). As an example, we confirmed the knockdown of one factor, Sin3a, at the protein level, validated its differential effects on the expression of set of chemokine genes and showed that Sin3a was recruited to chromatin. Ultimately, we identify 22 nuclear cofactors whose knockdown by several shRNAs suppresses or enhances expression of the target gene *Cxcl2* in the same direction thus reducing the likelihood of off-target effects (78). Accordingly, the involvement in gene regulation of 16 cofactors was reproduced by thorough RT-qPCR validation. Our data analysis procedure further reveals that basal mRNA expression is the most variable biological factor influencing screening results when searching for factors modulating highly regulated genes as the real time PCR procedures used here were sensitive, efficient and highly specific (Figure S10 in Supplementary Material). We suggest that special care should be taken to calculate shRNA-dependent changes in mRNA expression for basal and inducible conditions. Furthermore, our data provide several examples of shRNAs that lower or enhance constitutive and IL-1-inducible mRNA in the same direction. In this case, a regulatory role of the shRNA target gene can be easily missed if relative cytokine-induced changes are calculated separately against vector control and shRNA transfection. Our global analysis of mRNA expression also revealed that care should be taken to use GFP-expressing vectors as a control as our results clearly reveal a dominant negative effect on *Cxcl2* mRNA but also on *ActB* and *Ube2l3* expression. This is in line with recent studies showing that GFP inhibits IL-2 expression in T cells and that NF-κB-driven GFP reporter constructs deteriorate expression of LPS- or TNF-induced endogenous *IL6* and *Cxcl1* genes (79, 80). This might be due to defective polyubiquitinylation in GFP expressing cells which can perturb ubiquitin-based signaling cascades (81). In addition, free GFP has been reported to accumulate within the nucleus forming homotetramers or homohexamers (82). These GFP multimers might sequester chromatin complexes and cofactors that are required for endogenous regulation of genes, a phenomenon that might be missed in shRNA screens that use NF-κB-driven GFP reporter assays as global readouts for gene activation.

Further support for the strategy chosen here to identify relevant coactivators/corepressors for inflammatory gene expression can be derived from a limited number of published findings implicating these proteins in NF-κB activation or immune functions or by showing that their expression is regulated by TNF or IL-1 (Table 1). One example is the Clock TF that cooperates with the p65 NF-κB subunit to regulate the circadian expression of genes such as *Dbp1* and *Per1-3* (31, 83). These Clock target genes are downregulated in IL-1-or TNF-triggered cells (83). In turn, the repressive Clock target gene Cryptochrome suppresses activation of NF-κB suggesting that the circadian-oscillator components and immune pathways are tightly intertwined (84, 85). Our results support these findings and show that conversely, Clock is required for full activation of inflammatory genes in the IL-1 pathway. Another factor is *Rps6ka4* encoding the protein kinase MSK2. The closely related kinase MSK1 phosphorylates p65 NF-κB at S276 promoting its activation (60). Both kinases are also major histone H3S10 and CREB/ATF1 kinases and hence affect gene regulation at multiple levels (86). However, MSK1- and MSK2-double deficient mice show increased skin inflammation and activation of LPS target genes, an effect that was attributed to decreased levels of Dusp1 and IL-10, which are negative regulators of LPS signaling (61). Our results support a repressive and non-redundant role of MSK2 in inflammatory cell reactions by negative regulation of some but not all IL-1-target genes. The NCOA1 protein binds to the *Il6* and *Nfkbia* promoters, coactivates *Il6, Nfkbia*, and NF-κB promoter luciferase constructs (together with p65) and is needed for angiotensin-II-mediated *IL6* or for TNF-induced *Nfkbia* mRNA expression (52, 53). Here, loss of *Ncoa1* severely suppressed mRNA expression of six chemokine genes (except *Ccl7*), *Icam1* and *Il6* but was without any effect on *Nfkbia*. In contrast, knockdown of the related *Ncoa6* derepresses five chemokine genes plus *Il6* but suppresses expression of *Cxcl10*. The latter is in contrast to one study which found that NCOA6 weakly stimulates p65-driven activation of NF-κB reporters (54). Thus, the effects of both coactivators are highly gene specific and partially opposing. Also, overexpressed PRMT6 was shown in one report to enhance TNF-mediated NF-κB nuclear translocation and activation of a NF-κB luciferase reporter. However, despite global activation of NF-κB in these assays, the ectopically expressed PRMT6 protein only enhanced TNF-mediated mRNA induction of *Il6, Tnf*, and *COX2* (*Ptgs2*) but not *MCP1* (*Ccl2*) or *Nfkbia*. In ChIP assays, PRMT6 also bound to the *IL6* and *TNF* promoters together with p65 (59). This study nicely illustrates the different results obtained by using global reporter assays compared to investigating specific genes. In our screen, knockdown of PRMT6 enhanced *Cxcl2* expression, suggesting that for this gene PRMT6 is a suppressor rather than an activator. Altogether these comparisons reveal the necessity to study the true function of nuclear cofactors at the endogenous mRNA or protein level.

The majority of factors identified in our screen have no established role in inflammatory gene expression (Table 1). Although some of them such as *Sin3a, Padi4, Phf21a, Kdm6a, Ruvbl1*, or *Kdm5a* are powerful activators or repressors of most of these genes, the overall result is that these cofactors execute gene specific functions. The mechanistic basis for this phenomenon awaits further investigations but is in line with conclusions derived from other systems, in particular the nuclear hormone receptor complexes (9). It will also be interesting to find out if any of these effects are specific to IL-1. So far, the only cellular molecule that is specific for the IL-1 pathway is the IL-1 type I receptor. All other pathway components including the entire NF-κB pathway are shared by other signaling systems (4, 87, 88). The 22 molecules that form the IL-1 coactivator network comprise different functional categories. There are many genes encoding enzymes that phosphorylate proteins (*Dapk3, Rps6ka4*), methylate or acetylate histone residues (*Kdm5a, Kdm2b, Kdm6a, Prmt6, Sirt3, Suv420h1*), remodel chromatin (*Ruvbl1*) or deiminate or ubiquitylate histones (*Padi4, Ube3a*). Several other genes encode scaffolds or components of corepressor complexes (*Sin3a, Sin3b, Mbd3, Mta2, Phf21a, Epc1*) (summarized in Table 1). It is therefore possible that these factors are components of cofactor complexes which have been remodeled from classical coactivator/corepressor complexes such as the NuRD, Sin3a, or CoRest complexes into IL-1-specific nuclear factories. These multiprotein complexes are predicted to assemble at chromatin to coordinate and synchronize the genome-wide IL-1-gene response in space and time. According to this concept, the specificity of the inflammatory gene response is encoded in the nuclear compartment rather than in the cytosolic signaling pathways.

In conclusion, the data derived from these screening experiments give raise to multiple new hypotheses. Proving a direct role of each of the factors in inflammatory gene expression will require a complex set of follow up studies including chromatin immunoprecipitation (ChIP) experiments. These experiments will be challenging as nuclear cofactors do not bind directly to DNA (unlike TFs) and are thus notoriously more difficult to crosslink and to immunoprecipitate. The cofactors may bind to distal enhancers or promoters or may regulate their target genes indirectly. Thus, ChIP-seq experiments are needed to determine their true sites of chromatin recruitment across the genome, provided suitable ChIP-grade antibodies are available. Further, there is no requirement for dynamic or IL-1-dependent recruitment of cofactors to execute their functions as regulation of their activity might occur entirely through posttranslational modifications. All these experiments are beyond the scope of our study, which reports new functional evidence derived from shRNA experiments.

## Materials and Methods

### Cells and Cell Culture Materials

Mouse embryonic fibroblasts (Mef line 1, TA7) immortalized by the 3T3 protocol and carrying targeted alleles allowing conditional Cre-mediated deletion of HDAC3 (Hdac3^fl/−^) were used for the shRNA screens and for all RT-qPCR validation experiments (89). These cells have normal HDAC3 protein levels in the absence of Cre activation and their IL-1 response has been extensively characterized in a previous study from our lab (27). These cells were cultured in Dulbecco’s modified Eagle’s medium (DMEM), complemented with 10% fetal calf serum, 2 mM l-glutamine, 1× non-essential amino acids, 100 U/ml penicillin and 100 µg/ml streptomycin. p65 NF-κB-deficient (Mefs p65^−/−^), reconstituted Mefs (p65^−/−^ + p65 wt) (90) and Mef line 2 (MK2/5) (91) were cultured in Dulbecco’s modified Eagle’s medium (DMEM), complemented with 10% fetal calf serum, 2 mM l-glutamine, 100 U/ml penicillin, and 100 µg/ml streptomycin.

### Plasmid Purification and Transfections for the shRNA Screens and Validation Experiments

For the shRNA screens and validation experiments, mouse shRNA vectors, derived from TRC.1 library^1^ as glycerol stocks, were purified by the PureYield™ plasmid miniprep system from Promega following the manufacturer’s instructions. pLKO.1-puro empty vector, pLKO.1-puro non-mammalian shRNA (scrambled shRNA) vector, and pLKO.1-puro GFP vector were used as negative controls or for transfection efficiency control by fluorescence microscopy. For transient transfection screening, 3.5 × 10^4^ Hdac3^fl/−^ Mefs were seeded in 48-well plates. 270 ng plasmid DNA per well was transfected using Lipofectamine^®^ LTX and Plus^TM^ reagent from Invitrogen^TM^ following the manufacturer’s instructions. 24 h post transfection, transfected cells were selected for 48 h by adding 1 µg/ml puromycin. For larger scale RT-qPCR validation experiments, 4 × 10^5^ Hdac3^fl/−^ MEFs were seeded in 60-mm cell culture dishes and transfected with 5.5 µg plasmid DNA. After puromycin selection cells were left untreated or were treated with human recombinant IL-1α (10 ng/ml) (kind gift from Jeremy Saklatvala, London, UK) for the indicated periods of time.

### Measurements of mRNA Expression

mRNA measurements were performed by conventional RT-qPCR or Agilent microarrays as described before (27, 92). For the shRNA screens, cDNA was synthesized in cell lysates and amplified using the TaqMan^TM^ PreAmp Cells-to-Ct Kit^TM^ and TaqMan^®^ Gene-Expression Assays from Applied Biosystems^TM^ following an adapted miniaturized protocol. The kit enables to perform gene expression analysis directly from limited or small numbers of cultured cells without RNA purification by including an intermediate amplification step (pre-amplification) between reverse transcription and real-time PCR. Cells were washed twice in cold PBS, transferred to tubes, and lysed in 12.5 µl lysis solution (DNase I was diluted at 1:100). After mixing five times the lysates were incubated for 5 min at room temperature. The reaction was stopped by adding 1.25 µl stop solution. After mixing five times, the samples were incubated for 2 min at room temperature. For reverse transcription, 4.5 µl of the lysate was used for a final reaction volume of 10 µl. 5 µl of 2× RT-buffer and 0.5 µl of 20× RT enzyme mix were added. The reaction tubes were incubated in a thermal cycler at 37°C for 60 min, then at 95°C for 5 min to inactivate the RT enzyme. In the following step, the cDNA was amplified using gene-specific primers contained in TaqMan^®^ gene expression assays. The assays of interest were diluted 1:100 in 1× TE and 2.5 µl were used in a 10 µl reaction volume with 2.5 µl cDNA and 5 µl 2× TaqMan PreAmp MasterMix. The preamplification occurred in a thermal cycler at 95°C for 10 min, following 15 cycles at 95°C for 15 s/60°C for 4 min. Prior to real-time PCR, the preamplification products were diluted 1:5 with 1× TE. The following TaqMan^®^ gene expression assays were used in this study: *mUbe2l3* (Mm00784559_s1), *mActB* (Mm00607939_s1), *mCxcl1* (Mm00433859_m1), *mCxCl2* (Mm00436450_m1), *mCxCl3* (Mm01701838_m1), *mCxCl5* (Mm00436451_g1), *mCxCl10* (Mm00445235_m1), *mIl6* (Mm00446190_m1), *mCcl1* (Mm00441236_m1), *mCcl2* (Mm00441242_m1), *mCcl7* (Mm00443113_m1), *mCcl11* (Mm00441238_m1), *mCcl20* (Mm00444228_m1), *mNfkbiz* (Mm00600514_m1), *mNfkbia* (Mm00477798_m1), *mCar13* (Mm00517925_m1), *mMcm7* (Mm0083349_g1), *mEvl* (Mm00468405_m1), and *mIcam1* (Mm00516023_m1). The expression of the indicated target genes was determined by real-time PCR using the TaqMan^®^ Fast universal PCR master mix and 7500 Fast Real-Time PCR System from Applied Biosystems. The following mRNAs were detected by Fast SYBR^TM^ Green Master Mix: *mSin3a* (se: gccctgtcctatcttgacca; as: ttttgtagccaggaggcaag) and *mMbd3* (se: ggccacagggatgtctttta; as: ttgcttgaagatggatgcag). Representative examples for amplification and melting curves are provided in Figure S10 in Supplementary Material indicating the efficiency and specificity of PCR reactions.

Relative changes of mRNA expression compared to the unstimulated pLKO.1 vector control were normalized to the expression of *mUbe2l3* and quantified using the 2^−ΔΔCt^ method. Further ratio comparisons were calculated as shown in Figure S3 in Supplementary Material.

### Bioinfomatics and Calculations

Calculations, graphical representations, and statistical tests of data were performed using SigmaPlot11 and MS EXCEL2010. Z-scores were calculated according to the formula: *z* = (*x* − μ(mean))/σ(SD). Concerning box plots, the boundary of the box closest to zero indicates the 25th percentile, the line within the box marks the median, and the boundary of the box farthest from zero indicates the 75th percentile. Whiskers (error bars) above and below the box indicate the 90th and 10th percentiles. Points mark the remaining outliers. The STRING database and Cytoscape were used for network analysis and visualizations (30, 93). 18 non-regulated “housekeeping genes” were taken from RNA-seq data of 15 mouse tissues (94). For these and the ten NF-κB target genes (*Ccl2, Ccl7, Cxcl1, Cxcl10, Cxcl2, Cxcl5, Icam1, Il6, Nfkbia, Nfkbiz*), 1000 bp of flanking DNA sequences were batch-extracted from the Ensembl database of the *Mus musculus* genome version GRCm38.p5 using Biomart software.^2^ Genomic sequences were searched for high quality NF-κB matrices using Match^TM^ public 1.0 from TRANSFAC® public 6.0 database (95).

### Chromatin Immunopecipitation

2 × 175-cm^2^ flask of confluent MEF cells, untreated or treated for 1 h with IL-1α (10 ng/ml), was used for each condition. Proteins bound to DNA were cross-linked *in vivo* with 1% formaldehyde added directly to the medium. After 10 min incubation at room temperature, 0.1 M glycine was added for 5 min to stop the cross-linking. Then, cells were collected by scraping and centrifugation at 1,610×*g* (5 min, 4°C), washed in cold PBS containing 1 mM PMSF and centrifuged again at 1,610×*g* (5 min, 4°C). Cells were lysed for 10 min on ice in 3 ml ChIP lysis buffer (1% SDS, 10 mM EDTA, 50 mM Tris pH 8.1, 1 mM PMSF, Roche protease inhibitor mix). The DNA was sheared by sonication (7 × 30 s on/30 s off, four times; Bioruptor, Diagenode) and lysates cleared by centrifugation at 16,100×*g* at 4°C for 15 min. Supernatants were collected and stored in aliquots at −80°C for subsequent ChIP. For determination of DNA concentration 20 µl of sheared lysate was diluted with 100 µl TE buffer including 10 µg/ml RNAse A. After 30 min at 37°C, 3.8 µl proteinase K (20 mg/ml) and 1% SDS were added and incubated for at least 2 h at 37°C followed by overnight incubation at 65°C. Samples were resuspended in two volumes of buffer NTB (Macherey & Nagel) and DNA was purified using Nucleo Spin columns (Macherey & Nagel) according to the manufacturer’s instructions. DNA was eluted with 50 µl 5 mM Tris pH 8.5, and concentration was determined by Nano Drop. For ChIP, the following antibodies were used: anti-NF-κB p65 (3 µg, Santa Cruz; sc-372), anti-RNA Pol II (1 µg, Millipore; 17-620), anti-P(S5)-Pol II (2 µg, Abcam; ab5131), anti-Sin3a (3 µg, Santa Cruz; sc-994), and IgG (2 µg, Cell Signaling; 2729). Antibodies were added to precleared lysate volumes equivalent to 25 µg of chromatin. Then, 900 µl of ChIP dilution buffer (0.01% SDS, 1.1% Triton X-100, 1.2 mM EDTA, 167 mM NaCl, 16.7 mM Tris/HCl pH 8.1) were added, and the samples were rotated at 4°C overnight. Thereafter, 30 µl of a protein A/G sepharose mixture, pre-equilibrated in ChIP dilution buffer was added to the lysates and incubation continued for 2 h at 4°C. Beads were collected by centrifugation, washed once in 900 µl ChIP low salt buffer (0.1% SDS, 1% Triton X-100, 2 mM EDTA, 20 mM Tris pH 8.1, 150 mM NaCl), once in 900 µl ChIP high salt buffer (0.1% SDS, 1% Triton X-100, 2 mM EDTA, 20 mM Tris pH 8.1, 500 mM NaCl), once in 900 µl ChIP LiCl buffer (0.25 M LiCl, 1% NP40, 1% desoxycholate, 1 mM EDTA, 10 mM Tris pH 8.1) and twice in 900 µl ChIP TE buffer (10 mM Tris pH 8.1, 1 mM EDTA) for 5 min at 4°C. Beads were finally resuspended in 100 µl TE buffer including RNase A (10 mg/ml). In parallel, 1/10 volume (2.5 µg) of the initial lysate (input samples) were diluted with 100 µl TE buffer including 10 µg/ml RNase A. After 30 min at 37°C, 3.8 µl proteinase K (20 mg/ml) and 1% SDS were added and both input and immunoprecipitates were incubated for at least 2 h at 37°C followed by overnight incubation at 65°C. Samples were resuspended in two volumes of buffer NTB (Macherey & Nagel) and DNA purified using Nucleo Spin columns (Macherey & Nagel) according to the manufacturer’s instructions. DNA was eluted with 50 µl 5 mM Tris pH 8.5 and stored at −20°C until further use.

### Quantification of ChIP DNA by Real-Time PCR

PCR products derived from ChIP were quantitated by real time PCR using the Fast ABI 7500 instrument (Applied Biosystems). The following primers were used as described in Ref. (29): Murine *Ccl2* promoter (sense “cgagggctctgcacttactc” & antisense “tcagtgagagttggctggtg”), *Ccl2* enhancer (sense “cccatgagagaactgcttgg” & antisense “ggcaggtcagaggcagagta”) and gene-free negative control region upstream of *Cxcl5* (sense “tttcatgcctctgagtgtgc” & antisense “ttttccctggctttgaccta”). The reaction mixture contained 2 µl of ChIP or input DNA (diluted 1:10 to represent 1% of input DNA), 0.25 µM of primers and 10 µl of Fast Sybr Green Mastermix (2×) (Applied Biosystems) in a total volume of 20 µl. PCR cycles were as follows: 95°C (20 s), 40× (95°C [3 s], 60°C [30 sec]). Melting curve analysis revealed a single PCR product. Calculation of enrichment by immunoprecipitation relative to the signals obtained for 1% input DNA was performed according to the following equation: percent of input = 2^−(Ct sample−Ct input)^.

### Immunoblotting

Preparation of total cell extracts and immunoblotting was performed as described (92). The following antibodies were used: anti SIN3A (Abcam, ab3479, 1:2,000 in TBS/0.05% Tween, 5% BSA), and anti tubulin (Santa Cruz, sc-8035, 1:1,000 in TBS/0.05% Tween, 5% milk).

## Author Contributions

JM-S and DN performed experiments, JM-S, AW, and MK analyzed data. JK and TB provided the shRNA library. LJ contributed to ChIP experiments. JM-S and MK conceived the study. MK wrote the initial draft. MS, JK, JM-S, and MK finalized the manuscript. All authors approved the submitted version of the manuscript.

## Conflict of Interest Statement

The authors declare that the research was conducted in the absence of any commercial or financial relationships that could be construed as a potential conflict of interest.

## Footnotes

^1^https://portals.broadinstitute.org/gpp/public/

^2^http://www.ensembl.org

## Supplementary Material

Figure S1 Selection of IL-1 target genes and housekeeping genes in murine embryonic fibroblasts used in this study. **(A)** The 30 most strongly regulated IL-1-response genes of the immortalized fibroblast cell line used in this study were extracted from two previously published microarray experiments (27). Fold changes compared to untreated cells are presented as heatmap and are ordered according to mean regulation. Green colors represent genes selected for further analysis. **(B)** Displayed in the lower graph is the mRNA expression (as raw cycle threshold [ct] values) of five non-regulated housekeeping genes obtained using samples from untreated and IL-1 treated fibroblasts. Fold changes determined for *Cxcl2* and *Ccl2* are shown in the upper graph for comparison. Shown are mean values ± SEM from a technical replicate.

Figure S2 Validation and kinetics of IL-1-mediated gene regulation of the immortalized murine embryonic fibroblast lines used in this study. Mef cells were stimulated for different times with IL-1 or were left untreated and expression of the indicated genes was analyzed by RT-qPCR. Shown are mean relative fold changes ± SEM from two independent experiments. Blue colors indicate seven chemokine genes. Red colors mark the two time points chosen for the shRNA screens.

Figure S3 Calculation of relative differences in mRNA expression across various biological conditions from cycle threshold (ct) values. The graph shows a hypothetical representation of *Cxcl2* mRNA levels in the absence or presence of IL-1 in vector control cells or in cells after shRNA-mediated knockdown. Numbers in circles refer to the equations used to calculate relative differences (fold changes) in basal or IL-1-induced expression levels or in the IL-1-mediated regulation comparing vector controls with each of the shRNA samples.

Figure S4 Heatmaps and ratio values of nuclear cofactors that repress or activate basal or inducible mRNA expression levels of *Cxcl2*. The tables and heatmaps show the computed fold changes for all shRNA samples of screen I and screen II that resulted in a Z-score below −1 or above 1 SD in the analyses shown in Figures 2C,D in both screens. The vector control values from the corresponding plates are shown for comparison. Colors indicate the direction of the shRNA effect (blue suppression, red induction of mRNA levels) or IL-1-triggered expression in vector controls.

Figure S5 Gene-specific requirements of the 16 nuclear cofactors for *Cxcl2* mRNA expression. Murine embryonic fibroblast (Mef) cells were transfected with pooled shRNAs directed against 16 nuclear cofactors identified by the shRNA screens shown in Figures 1 and 2. After 48 h of selection in puromycin, cells were treated with IL-1 for 1 h or were left untreated. Then, total RNA was isolated and mRNA expression of *Cxcl2* and *Ube2l3* was determined by conventional RT-qPCR. Expression values of *Cxcl2* were normalized for expression of *Ube2l3*. The graph shows relative expression values from two independent series of experiments (series 1 lanes 1–10, series 2 lanes 11–18). For each series all vector control values from IL-1-treated samples were pooled and set as 100%. Bars of shRNA samples represent mean values of at least three independent experiments with exception of *shEpc1* values which were derived from a biological duplicate experiment. Errors represent SEM. Controls of experiment series 1 (vector (1)) represent nine independent experiments, controls of experiment series 2 (vector (2)) represent three independent experiments. All data shown in Figures 3B, 4 and 5 and Figures S5–S7 in Supplementary Material are derived from these RNA preparations and the resulting ct values. Red or blue colors indicate at least 1.5-fold differences between shRNA versus empty vector transfections.

Figure S6 Gene-specific requirements of the 16 nuclear cofactors for 6 additional chemokine genes. The same RNA preparations described in Figure S5 were examined for the expression of additional chemokine genes by conventional RT-qPCR. Expression values were normalized for expression of *Ube2l3*. The graphs show relative expression values from two independent series of experiments (series 1, lanes 1–10; series 2, lanes 11–18). For each series, all vector control values from IL-1-treated samples were pooled and set as 100%. Bars of shRNA samples represent mean values of at least three independent experiments, *shEpc1* experiments represent duplicates. Red or blue colors indicate at least 1.5-fold differences between shRNA versus empty vector transfections.

Figure S7 Gene-specific requirements of the 16 nuclear cofactors for 4 additional IL-1 target genes. The same RNA preparations described in Figure S5 in Supplementary Material were examined for the expression of additional genes representing adhesion molecules, cytokines or regulators of the NF-κB pathway by conventional RT-qPCR. Expression values were normalized for expression of *Ube2l3*. The graphs show relative expression values from two independent series of experiments (series 1, lanes 1–10; series 2, lanes 11–18). For each series, all vector control values from IL-1-treated samples were pooled and set as 100%. Bars of shRNA samples represent mean values of at least three independent experiments, *shEpc1* experiments represent duplicates. Red or blue colors indicate at least 1.5-fold differences between shRNA versus empty vector transfections.

Figure S8 Essential function of p65 NF-κB in basal and inducible expression of IL-1 target genes. **(A)** The heatmap shows results from two microarray experiments comparing p65-deficient cells (p65^−/−^) with knockout cells reconstituted with p65 NF-κB as previously published by us (27). Fold changes after 1 h of treatment with IL-1 compared to untreated cells are presented as heatmap and are ordered according to mean regulation. Green colors represent genes investigated in this study.

Figure S9 Recruitment of SIN3A to the murine *Ccl2* locus. **(A)** Schematic representation of the regulatory regions of the murine *Ccl2* locus as previously published (29) and the regions amplified by PCR in chromatin immunoprecipitation (ChIP) reactions. **(B)** Murine embryonic fibroblasts (Mefs) used for all experiments shown in this study (TA7, Mef line 2) and an additional immortalized Mef line (MK2/5, Mef line 1) were stimulated for 1 h with IL-1 or were left untreated. Chromatin was prepared and recruitment of p65 NF-κB, Ser5-phosphorylated or non-phosphorylated RNA polymerase (Pol II) or SIN3A to two different regions of the *Ccl2* locus and a gene-free control region upstream of *Cxcl5* was examined by ChIP-PCR. IgG ChIP-PCRs were performed as controls. Shown is the mean enrichment of DNA fragments ± SEM from two (Mef line 2) or three (Mef line 1) independent experiments.

Figure S10 Examples of real time PCR amplification curves for *Cxcl2, Ube2l3, Sin3a*, and *Mbd3*. Shown are all amplification plots for the PCR reactions performed for Figure 3A indicating the efficiency and the specificity of the RT-PCR procedures used in this study. No DNA indicates PCR reactions, in which the cDNA template was substituted with H_2_O as a negative control.

Table S1 Identifiers, annotations, and small-hairpin (sh)RNA sequences for the 170 nuclear cofactors examined in this study.

Table S2 Enriched NF-κB binding sites in eleven IL-1 target genes. Shown are NF-κB motifs in the 1000 basepair flanking sequences of the eleven IL-1 target genes compared to a set of non-regulated murine genes.
