# Supplementary material for: RNAi-Based Identification of Gene-Specific Nuclear Cofactor Networks Regulating Interleukin-1 Target Genes
Source: Front Immunol. 2018 Apr 27;9:775. doi: 10.3389/fimmu.2018.00775 (PMC5934416; doi:10.3389/fimmu.2018.00775)
Supplement: Figure S1 — Selection of IL-1 target genes and housekeeping genes in murine embryonic fibroblasts used in this study. (A) The 30 most strongly regulated IL-1-response genes of the immortalized fibroblast cell line used in this study were extracted from two previously published microarray experiments (27). Fold changes compared to untreated cells are presented as heatmap and are ordered according to mean regulation. Green colors represent genes selected for further analysis. (B) Displayed in the lower graph is the mRNA expression (as raw cycle threshold [ct] values) of five non-regulated housekeeping genes obtained using samples from untreated and IL-1 treated fibroblasts. Fold changes determined for Cxcl2 and Ccl2 are shown in the upper graph for comparison. Shown are mean values ± SEM from a technical replicate. [file presentation_1.PDF]

A

Gene	1	2	mean
Cxcl2	10.6	8.3	9.5
Cxcl1	6.0	5.3	5.7
Ccl20	5.7	4.4	5.1
Cxcl10	4.7	2.5	3.6
Ccl7	3.9	3.0	3.5
Ccl2	3.9	3.0	3.4
Tnf	4.4	2.0	3.2
Zfp36	3.4	2.9	3.2
Irf1	3.6	2.1	2.8
Serpine1	3.2	2.1	2.7
Nfkbiz	3.3	2.0	2.7
Tnfaip3	3.3	2.0	2.6
Ch25h	3.3	1.9	2.6
Birc3	3.0	2.0	2.5
Rnd1	3.1	1.7	2.4
Cxcl5	2.7	1.4	2.1
Nfkbia	1.8	2.0	1.9
Pde4b	2.1	1.6	1.9
Il6	2.3	1.4	1.8
Map3k8	2.3	1.3	1.8
Tlr2	2.5	1.0	1.8
B2m	2.1	1.4	1.8
Fos	1.8	1.7	1.7
Ier3	1.9	1.6	1.7
Icam1	2.5	0.8	1.6
Bcl10	1.9	1.3	1.6
Mapk6	2.0	1.2	1.6
1810011O10Rik	2.0	1.2	1.6
Btg2	1.7	1.3	1.5
Ccrl2	2.3	0.7	1.5

experiment	1	2	mean
IL-1 (0.5 h) / vector	+	+	+

mRNA fold change (log_2)

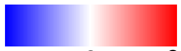

B

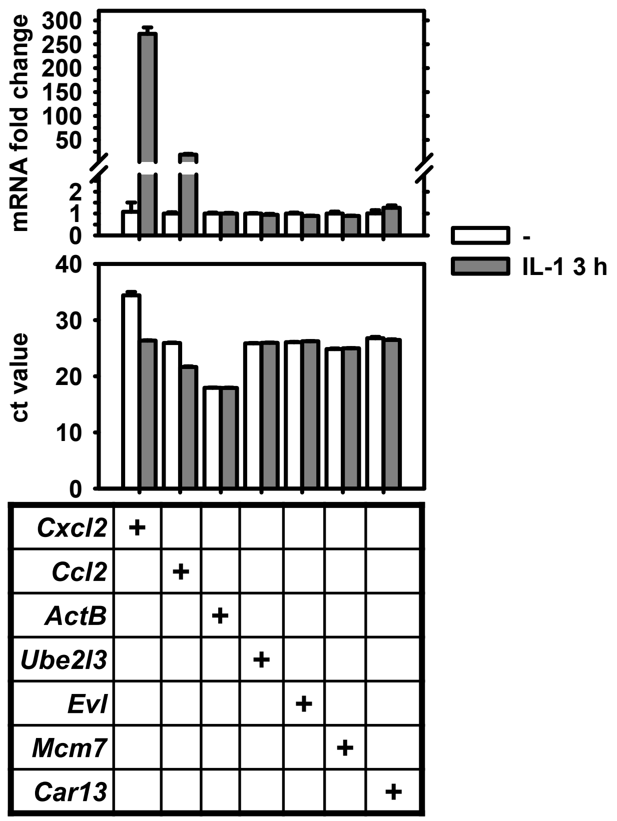

Fig.S1

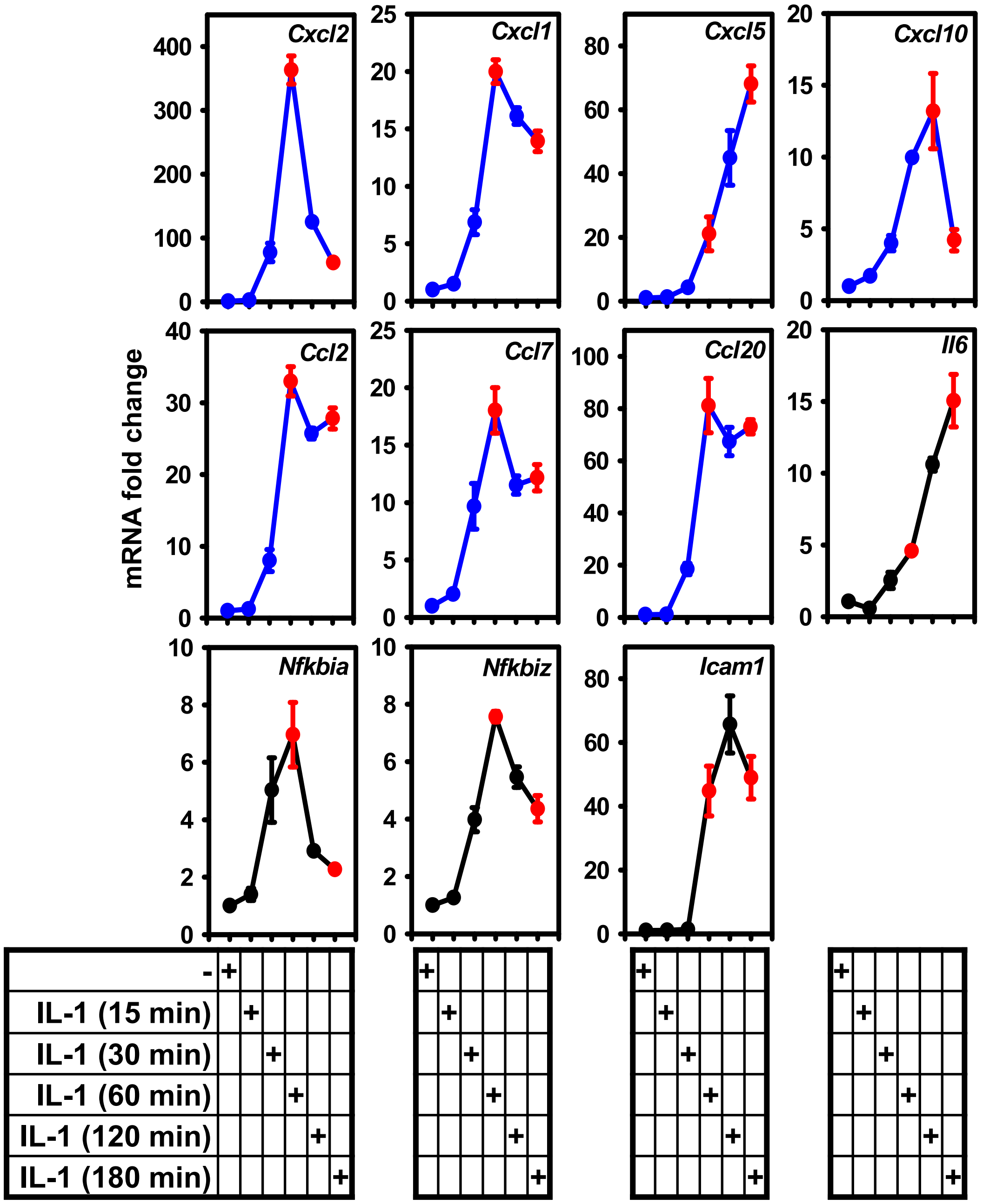
mRNA fold change
Cxcl2
Cxcl1
Cxcl5
Cxcl10
Ccl2
Ccl7
Ccl20
Il6
Nfkbia
Nfkbiz
Icam1
-
IL-1 (15 min)
IL-1 (30 min)
IL-1 (60 min)
IL-1 (120 min)
IL-1 (180 min)

Fig.S2

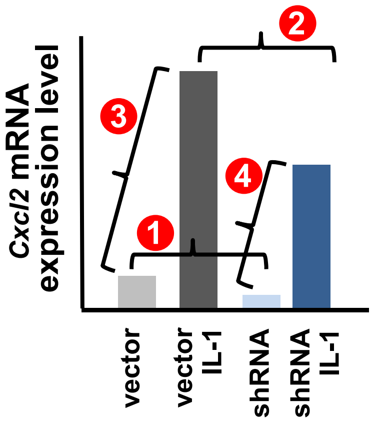

ratio comparisons to assess:

1 **basal expression levels**
2^- ΔΔct(shRNA)-(vector)

2 **IL-1-induced expression levels**
2^- ΔΔct(shRNA+IL-1)-(vector+IL-1)

3 **IL-1-mediated regulation in control cells**
2^- ΔΔct(vector+IL-1)-(vector)

4 **IL-1-mediated regulation in shRNA cells**
2^- ΔΔct(shRNA+IL-1)-(shRNA)

3/4 **differences in IL-1-mediated regulation**
2^- (ΔΔct(vector+IL-1)-(vector))-(ΔΔct(shRNA+IL-1)-(shRNA))

Fig.S3

shRNA effects on:

basal expression levels (ratio 1) (Z-score > 1 s.d.)

plate	gene	shRNA	fold change (shRNA / vector)	mean fold change	screen
4.0	*Sirt3*	*shSirt3 1*	0.08	0.01	I
4.0	*Sirt3*	*shSirt3 2*	0.01	0.01	I
4.0	*Sirt3*	*shSirt3 3*	0.01	0.01	I
4.0	*Sirt3*	*shSirt3 4*	0.01	0.01	I
4.0	*Sirt3*	*shSirt3 5*	0.01	0.01	I
7.0	*Taf1*	*shTaf1 1*	0.42	0.02	I
7.0	*Taf1*	*shTaf1 2*	0.00	0.02	I
7.0	*Taf1*	*shTaf1 3*	0.01	0.02	I
7.0	*Taf1*	*shTaf1 4*	0.25	0.02	I
7.0	*Taf1*	*shTaf1 5*	0.00	0.02	I
9.0	*Ncoa1*	*shNcoa1*	0.00	0.02	I
9.0	*Ncoa1*	*shNcoa1*	0.43	0.02	I
9.0	*Ncoa1*	*shNcoa1*	0.03	0.02	I
9.0	*Ncoa1*	*shNcoa1*	0.00	0.02	I
9.0	*Ncoa1*	*shNcoa1*	0.14	0.02	I
9.0	*Clock*	*shClock 1*	0.00	0.02	I
9.0	*Clock*	*shClock 2*	0.33	0.02	I
9.0	*Clock*	*shClock 3*	0.20	0.02	I
9.0	*Clock*	*shClock 4*	0.00	0.02	I
9.0	*Clock*	*shClock 5*	0.18	0.02	I
10.0	*Kdm2b*	*shKdm2b*	1.52	1.17	I
10.0	*Kdm2b*	*shKdm2b*	1.04	1.17	I
10.0	*Kdm2b*	*shKdm2b*	2.34	1.17	I
10.0	*Kdm2b*	*shKdm2b*	0.50	1.17	I
13.0	*Kdm6a*	*shKdm6a*	0.00	0.00	I
13.0	*Kdm6a*	*shKdm6a*	0.16	0.00	I
13.0	*Kdm6a*	*shKdm6a*	0.00	0.00	I
13.0	*Kdm6a*	*shKdm6a*	0.00	0.00	I
13.0	*Kdm6a*	*shKdm6a*	0.00	0.00	I
19.0	*Dapk3*	*shDapk3*	0.00	0.04	I
19.0	*Dapk3*	*shDapk3*	0.16	0.04	I
19.0	*Dapk3*	*shDapk3*	0.41	0.04	I
19.0	*Dapk3*	*shDapk3*	0.00	0.04	I
19.0	*Dapk3*	*shDapk3*	0.38	0.04	I
1.0	*Sirt3*	*shSirt3*	0.00		II
1.0	*Ncoa1*	*shNcoa1*	0.01		II
2.0	*Dapk3*	*shDapk3*	0.00		II
3.0	*Taf1*	*shTaf1*	0.00		II
3.0	*Clock*	*shClock*	0.00		II
5.0	*Kdm6a*	*shKdm6a*	0.00		II
6.0	*Kdm2b*	*shKdm2b*	5.64		II
			(vector+IL-1 / vector)		
4.0	*pLKO.1*		44.52	55.45	I
7.0	*pLKO.1*		40.39	55.45	I
9.0	*pLKO.1*		43.23	55.45	I
10.0	*pLKO.1*		183.88	55.45	I
13.0	*pLKO.1*		9.60	55.45	I
19.0	*pLKO.1*		16.79	55.45	I
1.0	*pLKO.1*		129.89	72.7	II
2.0	*pLKO.1*		101.89	72.7	II
3.0	*pLKO.1*		13.78	72.7	II
5.0	*pLKO.1*		83.00	72.7	II
6.0	*pLKO.1*		35.15	72.7	II

fold change

0.5 1 2

IL-1-induced expression levels (ratio 2) (Z-score > 1 s.d.)

plate	gene	shRNA	fold change (shRNA+IL1 / vector+IL1)	mean fold change	screen
12.0	*Kdm5a*	*shKdm5a 1*	2.30	1.23	I
12.0	*Kdm5a*	*shKdm5a 4*	0.37	1.23	I
12.0	*Kdm5a*	*shKdm5a 5*	2.18	1.23	I
13.0	*Kdm6a*	*shKdm6a 1*	0.09	0.18	I
13.0	*Kdm6a*	*shKdm6a 2*	0.24	0.18	I
13.0	*Kdm6a*	*shKdm6a 3*	0.48	0.18	I
13.0	*Kdm6a*	*shKdm6a 4*	0.26	0.18	I
13.0	*Kdm6a*	*shKdm6a 5*	0.06	0.18	I
15.0	*Suv420h*	*shSuv420h1*	0.01	0.16	I
15.0	*Suv420h*	*shSuv420h1*	0.67	0.16	I
15.0	*Suv420h*	*shSuv420h1*	0.63	0.16	I
15.0	*Suv420h*	*shSuv420h1*	0.20	0.16	I
18.0	*Phf21a*	*shPhf21a 1*	0.37	0.16	I
18.0	*Phf21a*	*shPhf21a 2*	0.13	0.16	I
18.0	*Phf21a*	*shPhf21a 3*	0.08	0.16	I
18.0	*Phf21a*	*shPhf21a 4*	0.26	0.16	I
18.0	*Phf21a*	*shPhf21a 5*	0.12	0.16	I
20.0	*Rps6ka4*	*shRps6ka4 1*	2.32	1.53	I
20.0	*Rps6ka4*	*shRps6ka4 2*	1.76	1.53	I
20.0	*Rps6ka4*	*shRps6ka4 3*	1.21	1.53	I
20.0	*Rps6ka4*	*shRps6ka4 4*	1.79	1.53	I
20.0	*Rps6ka4*	*shRps6ka4 5*	0.94	1.53	I
22.0	*Ube3a*	*shUbe3a 1*	1.78	1.18	I
22.0	*Ube3a*	*shUbe3a 2*	1.20	1.18	I
22.0	*Ube3a*	*shUbe3a 3*	1.08	1.18	I
22.0	*Ube3a*	*shUbe3a 4*	1.07	1.18	I
22.0	*Ube3a*	*shUbe3a 5*	0.92	1.18	I
24.0	*Sin3a*	*shSin3a 1*	0.16	0.16	I
24.0	*Sin3a*	*shSin3a 2*	0.07	0.16	I
24.0	*Sin3a*	*shSin3a 3*	0.27	0.16	I
24.0	*Sin3a*	*shSin3a 4*	0.49	0.16	I
24.0	*Sin3a*	*shSin3a 5*	0.07	0.16	I
24.0	*Sin3b*	*shSin3b 1*	0.15	0.22	I
24.0	*Sin3b*	*shSin3b 2*	0.14	0.22	I
24.0	*Sin3b*	*shSin3b 3*	0.62	0.22	I
24.0	*Sin3b*	*shSin3b 4*	0.15	0.22	I
24.0	*Sin3b*	*shSin3b 5*	0.27	0.22	I
26.0	*Ncoa6*	*shNcoa6 1*	0.84	1.18	I
26.0	*Ncoa6*	*shNcoa6 2*	0.58	1.18	I
26.0	*Ncoa6*	*shNcoa6 3*	1.00	1.18	I
26.0	*Ncoa6*	*shNcoa6 4*	3.08	1.18	I
26.0	*Ncoa6*	*shNcoa6 5*	1.50	1.18	I
27.0	*Ruvbl1*	*shRuvbl1 1*	2.01	1.87	I
27.0	*Ruvbl1*	*shRuvbl1 2*	0.86	1.87	I
27.0	*Ruvbl1*	*shRuvbl1 3*	1.33	1.87	I
27.0	*Ruvbl1*	*shRuvbl1 4*	5.34	1.87	I
29.0	*H2afz*	*shH2afz 1*	1.43	1.56	I
29.0	*H2afz*	*shH2afz 2*	0.97	1.56	I
29.0	*H2afz*	*shH2afz 3*	4.46	1.56	I
29.0	*H2afz*	*shH2afz 4*	1.39	1.56	I
29.0	*H2afz*	*shH2afz 5*	1.08	1.56	I
34.0	*Mta2*	*shMta2 1*	1.16	1.45	I
34.0	*Mta2*	*shMta2 2*	0.5	1.4	I
34.0	*Mta2*	*shMta2 3*	12.3	1.4	I
34.0	*Mta2*	*shMta2 4*	0.6	1.4	I
34.0	*Mta2*	*shMta2 5*	1.4	1.4	I
35.0	*Mbd3*	*shMbd3 1*	0.5	1.4	I
35.0	*Mbd3*	*shMbd3 2*	2.7	1.4	I
35.0	*Mbd3*	*shMbd3 3*	5.7	1.4	I
35.0	*Mbd3*	*shMbd3 4*	0.7	1.4	I
35.0	*Mbd3*	*shMbd3 5*	1.1	1.4	I
40.0	*Padi4*	*shPadi4 1*	0.3	0.1	I
40.0	*Padi4*	*shPadi4 2*	0.2	0.1	I
40.0	*Padi4*	*shPadi4 3*	0.0	0.1	I
40.0	*Padi4*	*shPadi4 5*	0.4	0.1	I
1.0	*Padi4*	*shPadi4*	0.1		II
2.0	*Sin3a*	*shSin3a*	0.0		II
2.0	*Phf21a*	*shPhf21a*	0.1		II
2.0	*Suv420h*	*shSuv420h1*	0.1		II
4.0	*Sin3b*	*shSin3b*	0.1		II
5.0	*Kdm6a*	*shKdm6a*	0.0		II
7.0	*Rps6ka4*	*shRps6ka4*	3.9		II
7.0	*Mbd3*	*shMbd3*	9.9		II
7.0	*Ncoa6*	*shNcoa6*	3.9		II
8.0	*Mta2*	*shMta2*	3.3		II
8.0	*Ube3a*	*shUbe3a*	7.8		II
8.0	*H2afz*	*shH2afz*	4.5		II
8.0	*Ruvbl1*	*shRuvbl1*	2.7		II
8.0	*Kdm5a*	*shKdm5a*	4.6		II
			(vector+IL-1 / vector)		
12.0	*pLKO.1*		22.0	55.4	I
13.0	*pLKO.1*		9.6	55.4	I
15.0	*pLKO.1*		55.8	55.4	I
18.0	*pLKO.1*		44.4	55.4	I
22.0	*pLKO.1*		6.2	55.4	I
24.0	*pLKO.1*		106.5	55.4	I
26.0	*pLKO.1*		41.0	55.4	I
27.0	*pLKO.1*		13.0	55.4	I
29.0	*pLKO.1*		49.3	55.4	I
34.0	*pLKO.1*		30.3	55.4	I
35.0	*pLKO.1*		28.7	55.4	I
40.0	*pLKO.1*		161.8	55.4	I
1.0	*pLKO.1*		129.9	89.6	II
2.0	*pLKO.1*		101.9	89.6	II
4.0	*pLKO.1*		196.1	89.6	II
5.0	*pLKO.1*		83.0	89.6	II
7.0	*pLKO.1*		18.0	89.6	II
8.0	*pLKO.1*		8.9	89.6	II

IL-1-mediated regulation (ratios 3 /4) (Z-score > 1 s.d.)

plate	gene	shRNA	fold change (vector+IL-1 / vector) / (shRNA+IL-1 / shRNA)	mean fold change	screen
4.0	*Sirt3*	*shSirt3 1*	0.49	0.07	I
4.0	*Sirt3*	*shSirt3 2*	0.19	0.07	I
4.0	*Sirt3*	*shSirt3 3*	0.02	0.07	I
4.0	*Sirt3*	*shSirt3 4*	0.54	0.07	I
4.0	*Sirt3*	*shSirt3 5*	0.00	0.07	I
7.0	*Taf1*	*shTaf1 1*	0.70	0.05	I
7.0	*Taf1*	*shTaf1 2*	0.01	0.05	I
7.0	*Taf1*	*shTaf1 3*	0.09	0.05	I
7.0	*Taf1*	*shTaf1 4*	0.29	0.05	I
7.0	*Taf1*	*shTaf1 5*	0.00	0.05	I
9.0	*Clock*	*shClock 1*	0.00	0.04	I
9.0	*Clock*	*shClock 2*	1.03	0.04	I
9.0	*Clock*	*shClock 3*	0.12	0.04	I
9.0	*Clock*	*shClock 4*	0.00	0.04	I
9.0	*Clock*	*shClock 5*	0.50	0.04	I
17.0	*Prmt6*	*shPrmt6 1*	5.74	5.17	I
17.0	*Prmt6*	*shPrmt6 2*	0.05	5.17	I
17.0	*Prmt6*	*shPrmt6 3*	20.54	5.17	I
17.0	*Prmt6*	*shPrmt6 4*	131.43	5.17	I
24.0	*Sin3b*	*shSin3b 1*	0.10	3.15	I
24.0	*Sin3b*	*shSin3b 2*	28.41	3.15	I
24.0	*Sin3b*	*shSin3b 3*	2.12	3.15	I
24.0	*Sin3b*	*shSin3b 4*	3.10	3.15	I
24.0	*Sin3b*	*shSin3b 5*	16.66	3.15	I
32.0	*Epc1*	*shEpc1 1*	0.1	0.1	I
32.0	*Epc1*	*shEpc1 2*	0.2	0.1	I
32.0	*Epc1*	*shEpc1 3*	0.0	0.1	I
32.0	*Epc1*	*shEpc1 4*	0.0	0.1	I
1.0	*Sirt3*	*shSirt3*	0.00		II
3.0	*Epc1*	*shEpc1*	0.00		II
3.0	*Taf1*	*shTaf1*	0.00		II
3.0	*Clock*	*shClock*	0.00		II
4.0	*Prmt6*	*shPrmt6*	3.40		II
4.0	*Sin3b*	*shSin3b*	5.07		II
			(vector+IL-1 / vector)		
4.0	*pLKO.1*		44.52	55.45	I
7.0	*pLKO.1*		40.39	55.45	I
9.0	*pLKO.1*		43.23	55.45	I
17.0	*pLKO.1*		126.31	55.45	I
24.0	*pLKO.1*		106.52	55.45	I
1.0	*pLKO.1*		129.9	113.2	II
3.0	*pLKO.1*		13.8	113.2	II
4.0	*pLKO.1*		196.1	113.2	II

Fig.S4

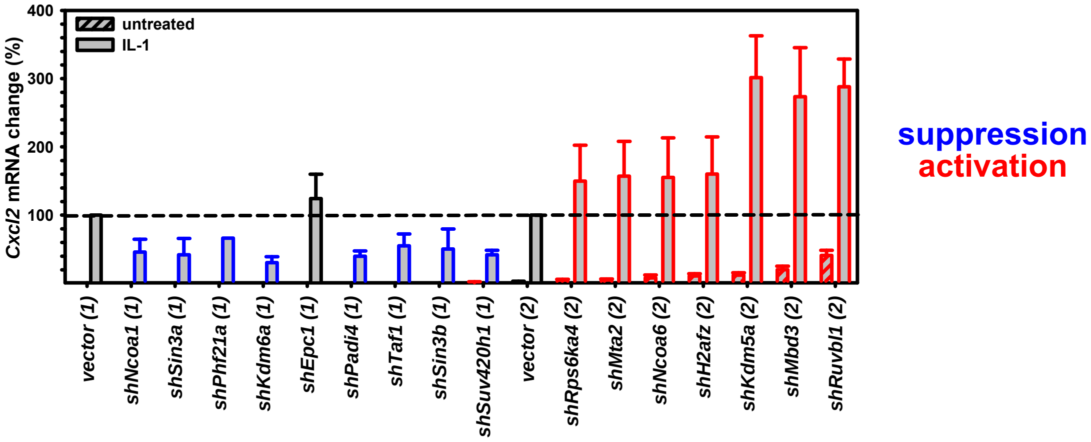
400
300
200
100
Cxcl2 mRNA change (%)
untreated
IL-1
vector (1)
shNcoa1 (1)
shSin3a (1)
shPhf21a (1)
shKdm6a (1)
shEpc1 (1)
shPadi4 (1)
shTaf1 (1)
shSin3b (1)
shSuv420h1 (1)
vector (2)
shRps6ka4 (2)
shMta2 (2)
shNcoa6 (2)
shH2afz (2)
shKdm5a (2)
shMbd3 (2)
shRuvbl1 (2)
suppression
activation

Fig.S5

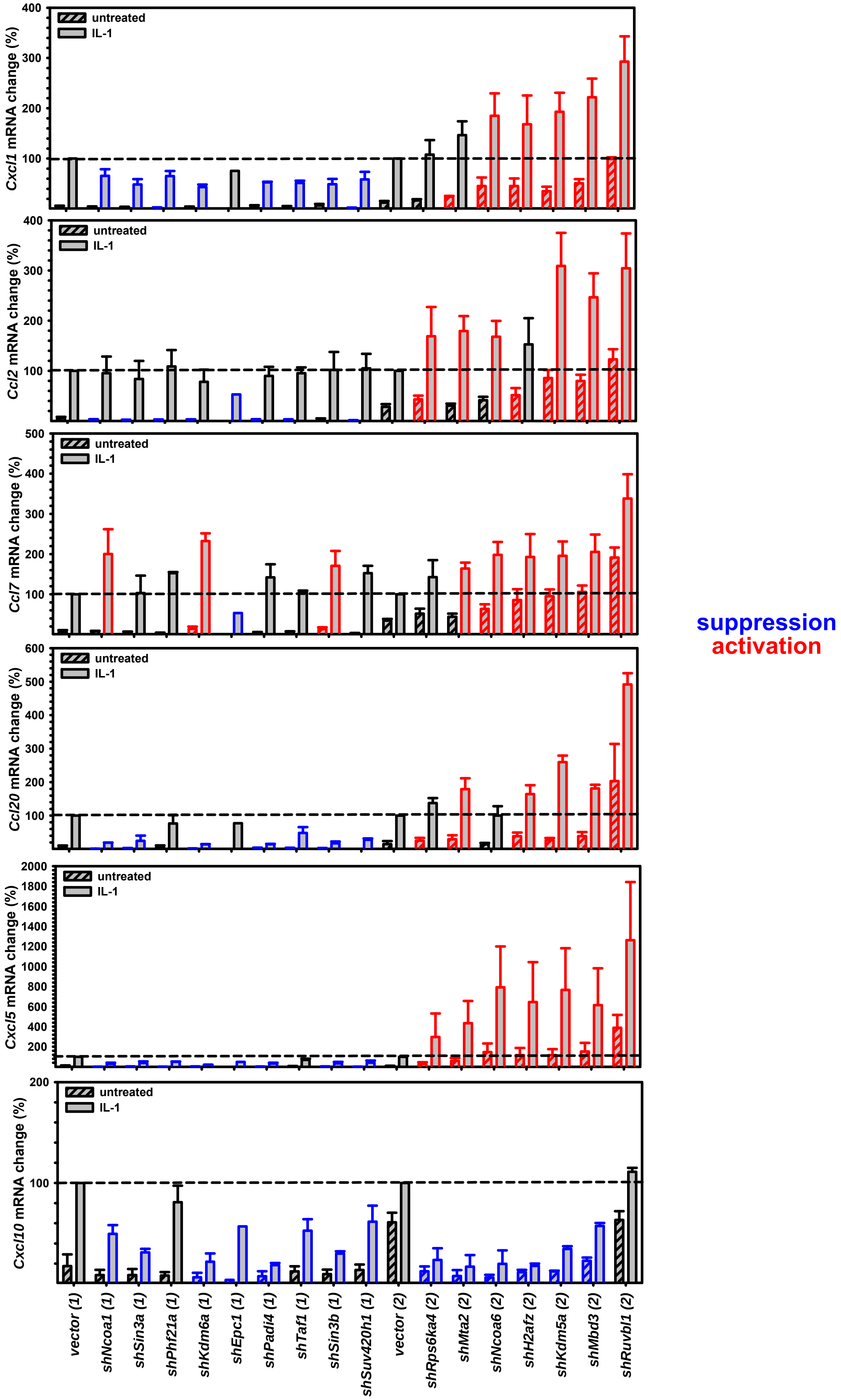

untreated
IL-1
Cxcl1 mRNA change (%)
Ccl2 mRNA change (%)
Ccl7 mRNA change (%)
Ccl20 mRNA change (%)
Cxcl5 mRNA change (%)
Cxcl10 mRNA change (%)
vector (1)
shNcoa1 (1)
shSin3a (1)
shPhf21a (1)
shKdm6a (1)
shEpc1 (1)
shPadi4 (1)
shTaf1 (1)
shSin3b (1)
shSuv420h1 (1)
vector (2)
shRps6ka4 (2)
shMta2 (2)
shNcoa6 (2)
shH2afz (2)
shKdm5a (2)
shMbd3 (2)
shRuvbl1 (2)
suppression
activation

Fig.S6

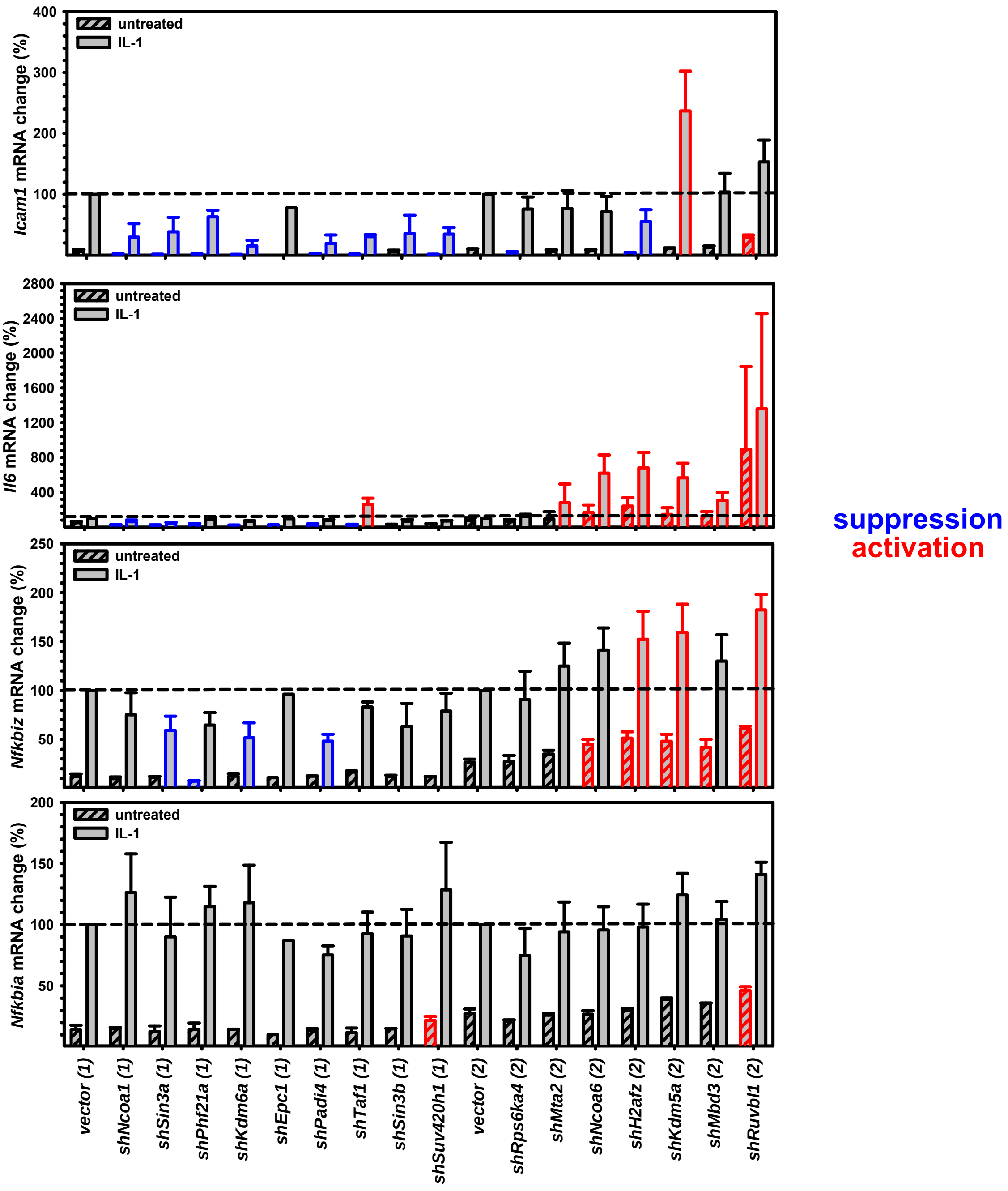
untreated
IL-1
Icam1 mRNA change (%)
Il6 mRNA change (%)
Nfkbiz mRNA change (%)
Nfkbia mRNA change (%)
suppression
activation
vector (1)
shNcoa1 (1)
shSin3a (1)
shPhf21a (1)
shKdm6a (1)
shEpc1 (1)
shPadi4 (1)
shTaf1 (1)
shSin3b (1)
shSuv420h1 (1)
vector (2)
shRps6ka4 (2)
shMta2 (2)
shNcoa6 (2)
shH2afz (2)
shKdm5a (2)
shMbd3 (2)
shRuvbl1 (2)

Fig.S7

ratios (against p65 $^{-/-}$ + p65 wt)

gene									
Cxcl2	-1.9	-0.4	0.0	0.0	-1.8	0.3	4.8	4.3	4.6
Icam1	0.0	0.1	0.0	0.0	0.2	0.7	5.2	3.7	4.5
Cxcl10	-2.2	-1.9	0.0	0.0	-0.3	-1.2	3.4	2.2	2.8
Il6	-1.3	-1.4	0.0	0.0	0.7	-1.5	2.6	1.1	1.8
Nfkbia	-0.9	-1.2	0.0	0.0	1.0	-0.2	2.2	1.4	1.8
Ccl2	-3.4	-5.3	0.0	0.0	-1.5	-4.9	1.8	1.1	1.5
Nfkbiz	-0.4	-0.5	0.0	0.0	1.1	0.8	1.5	1.4	1.5
Ccl7	-3.1	-4.8	0.0	0.0	-1.7	-4.8	1.8	1.0	1.4
Cxcl1	-0.3	-0.3	0.0	0.0	0.8	-0.3	1.2	1.2	1.2
Cxcl5	-2.8	-4.7	0.0	0.0	-2.5	-5.1	1.0	0.8	0.9
experiment	1	2	1	2	1	2	1	2	mean
p65 $^{-/-}$	+	+			+	+			
p65 $^{-/-}$ + p65 wt			+	+			+	+	+
IL-1 (1 h)					+	+	+	+	+

mRNA fold change (log_2)

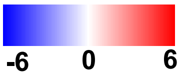

Fig.S8

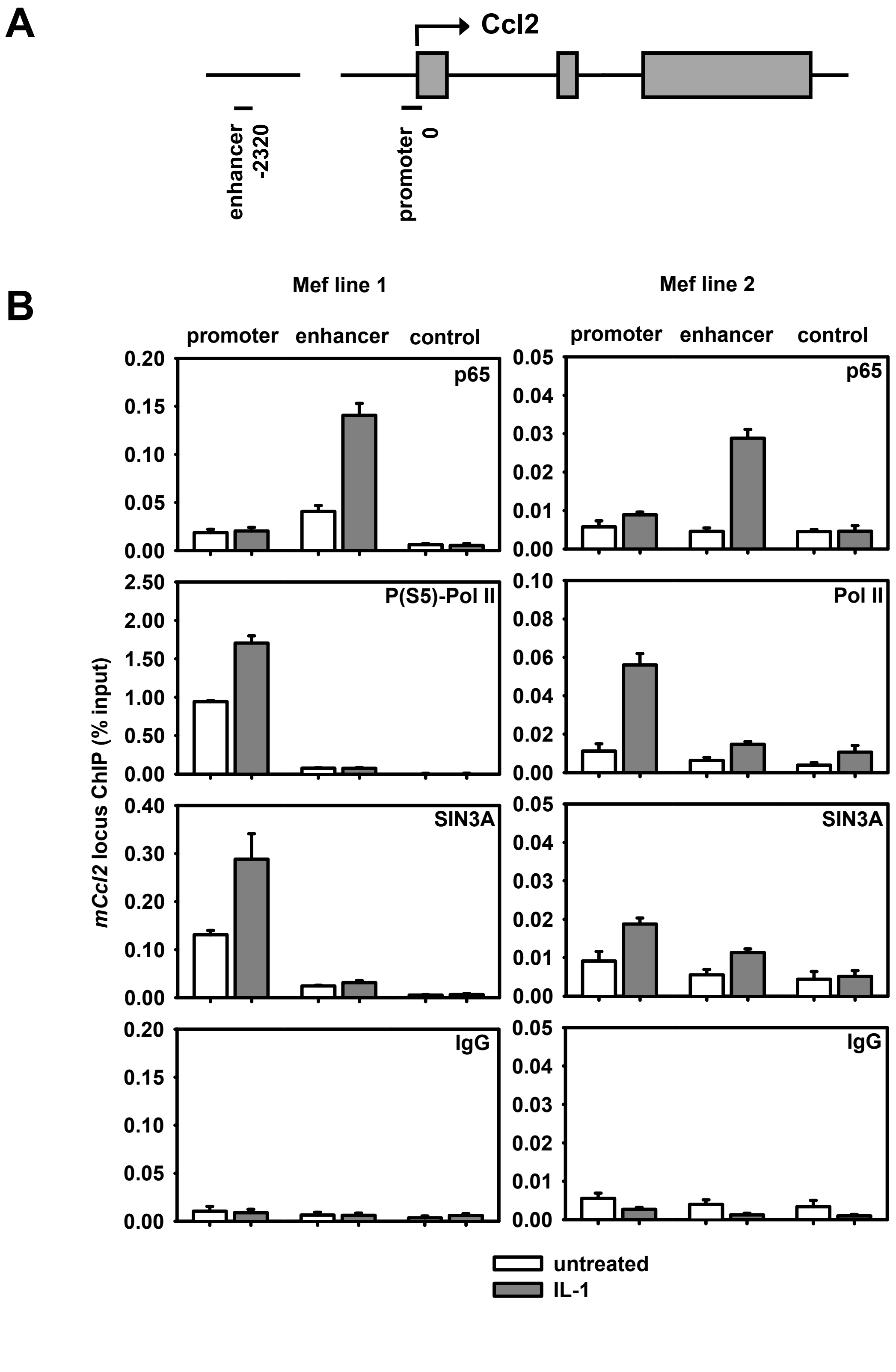
A
Ccl2
enhancer -2320
promoter 0
B
Mef line 1
Mef line 2
promoter
enhancer
control
p65
P(S5)-Pol II
Pol II
SIN3A
IgG
mCcl2 locus ChIP (% input)
untreated
IL-1

Fig.S9

PCR amplification curves using Taqman assays for *Cxcl2* and *Ube2l3*

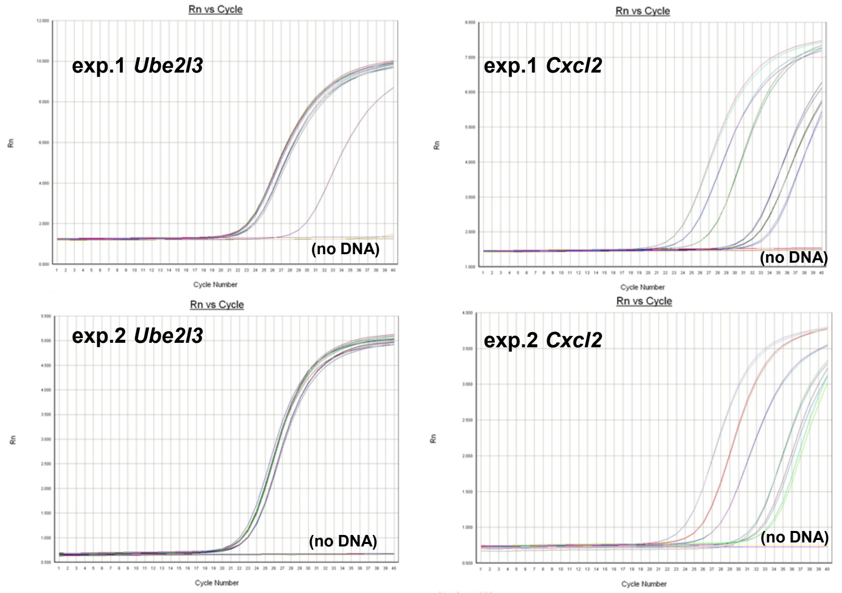

PCR amplification curves using Sybr Green assays for *Mbd3* and *Sin3a*

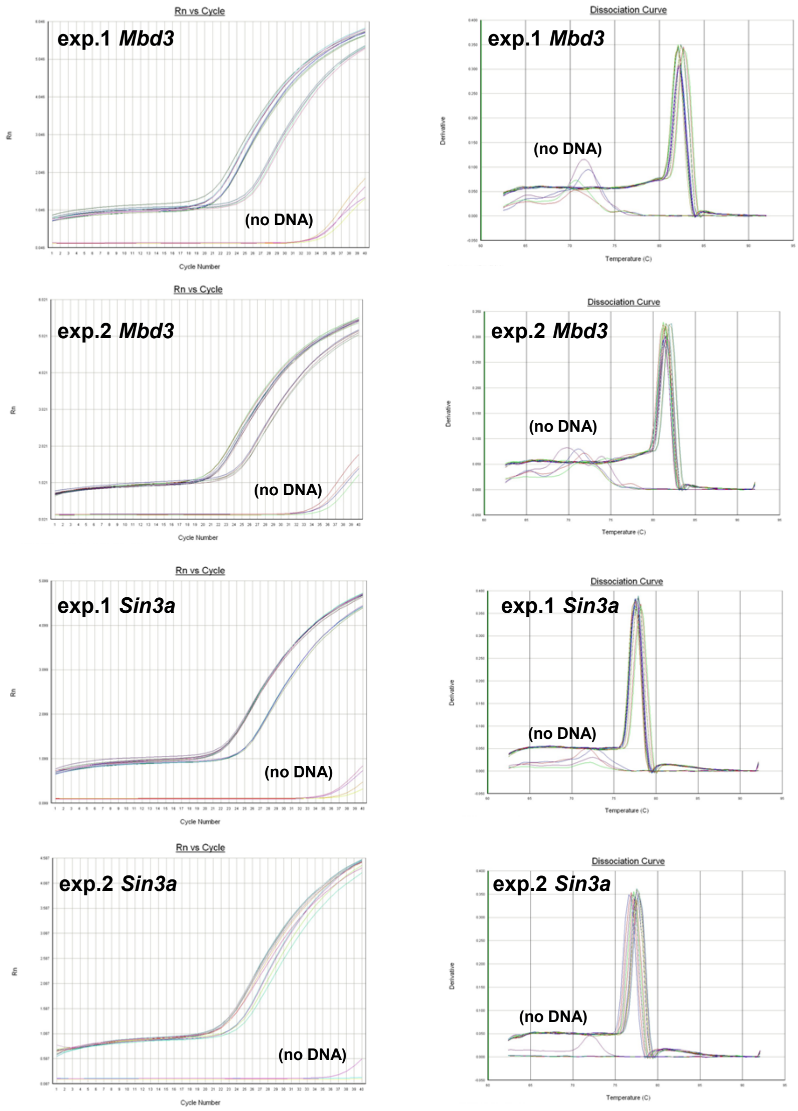

Fig.S10
